# How clinical imaging can assess cancer biology

**DOI:** 10.1186/s13244-019-0703-0

**Published:** 2019-03-04

**Authors:** Roberto García-Figueiras, Sandra Baleato-González, Anwar R. Padhani, Antonio Luna-Alcalá, Juan Antonio Vallejo-Casas, Evis Sala, Joan C. Vilanova, Dow-Mu Koh, Michel Herranz-Carnero, Herbert Alberto Vargas

**Affiliations:** 1 Department of Radiology, Hospital Clínico Universitario de Santiago de Compostela, Choupana s/n, 15706 Santiago de Compostela, Spain; 2 Paul Strickland Scanner Centre, Mount Vernon Cancer Centre, Northwood, Middlesex, England HA6 2RN UK; 3 Department of Radiology, University Hospitals of Cleveland, Case Western Reserve University, Cleveland, OH USA; 4 MRI Unit, Clínica Las Nieves, Health Time, Jaén, Spain; 5 Unidad de Gestión Clínica de Medicina Nuclear. IMIBIC. Hospital Reina Sofía. Universidad de Córdoba, Córdoba, Spain; 6 Department of Radiology and Cancer Research UK Cambridge Center, Cambridge, CB2 0QQ UK; 7 Department of Radiology, Clínica Girona and IDI, Lorenzana 36, 17002 Girona, Spain; 8 Department of Radiology, Royal Marsden Hospital & Institute of Cancer Research, Fulham Road, London, SW3 6JJ UK; 9 Nuclear Medicine Department, Hospital Clínico Universitario de Santiago de Compostela, Choupana s/n, 15706 Santiago de Compostela, Galicia Spain; 10 Molecular Imaging Program, IDIS, USC, Santiago de Compostela, Galicia Spain; 11 Department of Radiology, Memorial Sloan-Kettering Cancer Center, Radiology, 1275 York Av. Radiology Academic Offices C-278, New York, NY 10065 USA

**Keywords:** Neoplasms, Phenotype, Multimodal imaging

## Abstract

Human cancers represent complex structures, which display substantial inter- and intratumor heterogeneity in their genetic expression and phenotypic features. However, cancers usually exhibit characteristic structural, physiologic, and molecular features and display specific biological capabilities named hallmarks. Many of these tumor traits are imageable through different imaging techniques. Imaging is able to spatially map key cancer features and tumor heterogeneity improving tumor diagnosis, characterization, and management. This paper aims to summarize the current and emerging applications of imaging in tumor biology assessment.

**Electronic supplementary material:**

The online version of this article (10.1186/s13244-019-0703-0) contains supplementary material, which is available to authorized users.

## Key points

- Tumors demonstrate substantial inter- and intratumor heterogeneity in their biological features
- Imaging techniques may improve the assessment of tumor-specific characteristics in clinical practice.
- Functional and molecular imaging techniques may depict tumor heterogeneity.

## Introduction

**Fig. 1:**
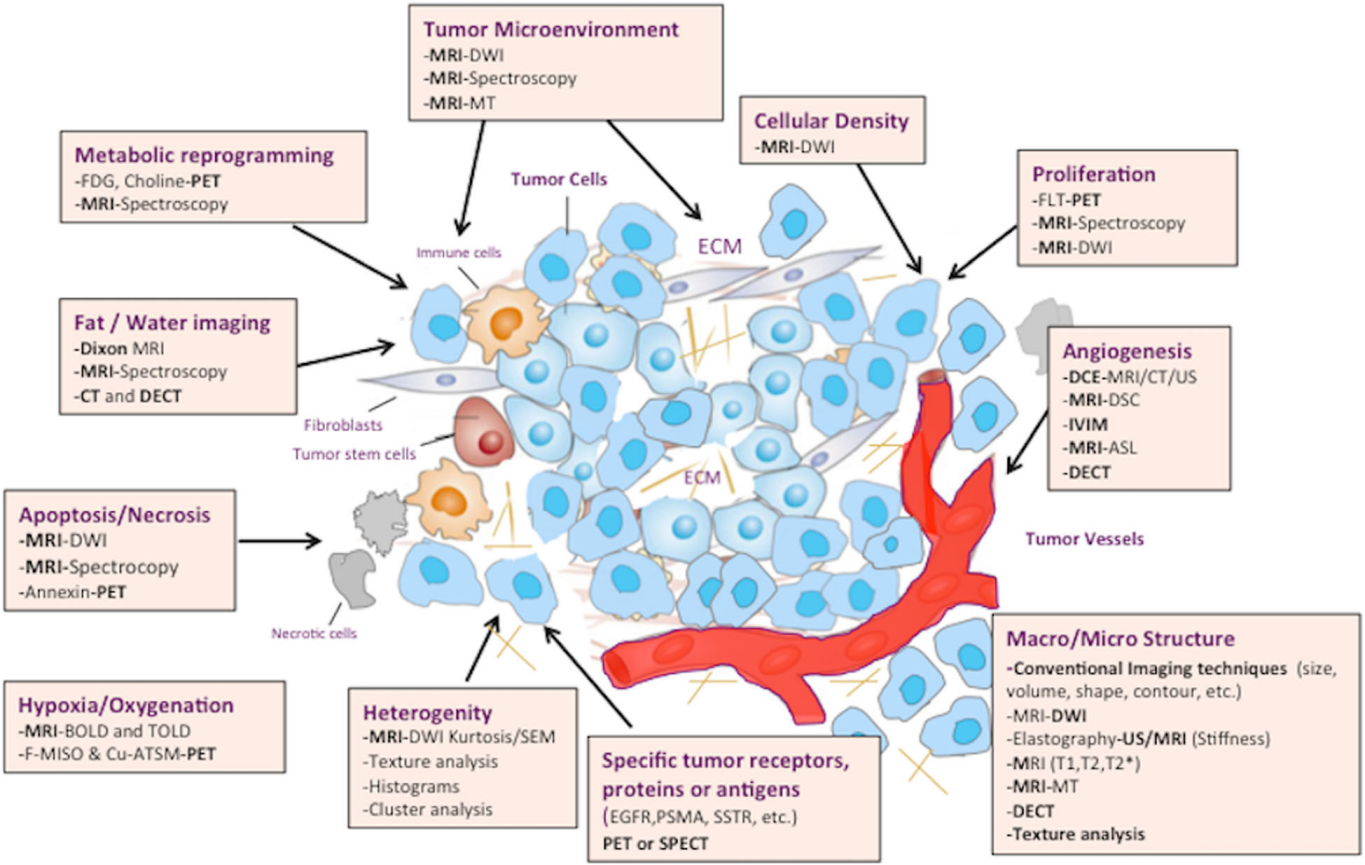
Main imaging techniques in the evaluation of tumor biology and microenvinroment

Imaging techniques have emerged as exceptionally powerful, versatile, and precise tools for assessing relevant tumor characteristics. Cancers usually display several structural, physiologic, and molecular changes and common acquired biological capabilities that can be evaluated with imaging [1, 2]. Besides, tumors demonstrate substantial inter- and intratumor heterogeneity in their biological features [3]. A modern personalized oncologic approach requires a deeper understanding of these cancer traits for being implemented in patient care. Functional-molecular imaging (FMI) information in addition to morphologic/anatomical changes can simultaneously assess a multitude of biological cancer-related processes, improving our diagnostic accuracy and the assessment of response to therapy [4]. Different imaging techniques are useful for this role, including dynamic contrast-enhanced MRI (DCE-MRI), DCE-ultrasound (US), dynamic susceptibility contrast-enhanced MRI (DSC-MRI), perfusion CT (PCT), diffusion-weighted imaging (DWI), and magnetic resonance spectroscopy (MRS) and spectroscopic imaging (MRSI), arterial spin-labeling (ASL), blood oxygenation level-dependent MR imaging (BOLD-MRI), elastography, positron emission tomography (PET), or single-photon emission computed tomography (SPECT) imaging (Fig. 1) (Additional file 1: Table S1). These imaging techniques enable the evaluation of unseen tumor characteristics by conventional techniques improving tumor diagnosis and management [4, 5]. In this ssetting, the biological and physiological correlations of imaging parameters (which depend on tumor type, imaging technique, and technical questions) need to be established and the limitations and possible pitfalls of every imaging technique must be asessed. This paper aims to summarize the role of imaging in the assessment of tumor biology.

## Imaging for the evaluation of tumor biology

Imaging techniques are increasingly being used to perform a noninvasive assessment of tumor biology in clinical practice. Imaging offers an adequate combination of anatomic, physiologic, and molecular information for evaluating cancer phenotype in vivo at different levels [6].

## Tumor macrostructural characteristics on imaging

Tumors are heterogeneous in nature and during malignant transformation, their structural features change substantially at different levels. Morphologic and structural differences between tumors are sometimes apparent and classical subjective descriptors of these characteristics (e.g., spiculated margin) are common. Tumor size and volume are usually important features in tumor evaluation and may have prognostic value. However, different imaging techniques may improve the assessment of tumor structure.

### Tumor morphology

The morphologic phenotype of neoplasms is widely variable; however, the presence of morphological features typical of benign, borderline, or malignant tumors increased the level of diagnostic confidence of radiologists. In this setting, different imaging-based classification systems (reporting and data system or -RADS) have been established to standardize imaging reporting and data collection in different organs, such as breast (BI-RADS), liver (LI-RADS), lung (Lu-RADS), thyroid (TI-RADS), etc. [7–10]. These -RADS lexicons include different morphological features (margin, shape, size, internal pattern, etc.) and provide an estimated risk of malignancy. So, well-defined tumor margins are considered an indicator of less infiltrative behavior and, thus, lower aggressiveness. Besides, a tumor morphologic phenotype may be associated to a pathologic entity. For instance, cystic change in renal cell carcinoma (RCC) has been mostly strongly associated with the clear cell subtype and most recent data indicate an overall favorable prognosis for the broad spectrum of predominantly cystic RCCs [11]. Finally, imaging features may be also associated to specific mutations. In the case of clear cell RCC, well-defined tumor margins were significantly more common among clear cell RCCs with loss of *von Hippel Lindau* (*VHL*) gene function [12]. The improvements in the computing capabilities of radiologic equipment have allowed the development of volume-based tumor measurements, which have demonstrated to be more sensitive to tumor changes, more reproducible and reliable than uni- or bi- dimensional measurements [13]. Tumor volume has a prognostic value in many tumor types and may improve tumor response evaluation [14]. Finally, tumor growth pattern also seems to be a prognostic significance in terms of overall survival. For example, the presence of a fibrous capsule and the characteristics of the tumor-liver interface are prognostically relevant in colorectal liver metastases [15].

### Biophysical characteristics of tumors: stiffness and elasticity

Solid tumors are typically stiffer than the surrounding healthy tissue. This feature is evaluated in clinical practice by palpation but elastographic imaging techniques can also assess the elastic properties and stiffness of tissues noninvasively.

Elastography provides knowledge about internal strains induced in a soft material undergoing an axial stress. There are two main imaging techniques for performing elastography: US and MRI [16, 17].

#### Technical features

The basic principle of elastography is to image the propagation of mechanical waves within the tissue. Stiffness, a biomechanical property of tissue, represents its ability to resist deformation when subjected to a force. Elastographic techniques evaluate these properties by a sequential process with three basic steps: (a) excitation (stress) based on the application of mechanical waves for deforming the tissue; (b) evaluating the tissue response (strain); and (c) assessment of stiffness and stiffness-related parameters displayed as quantitative parametric maps (elastograms) [16, 17]. Main elastographic techniques are based on the evaluation of the shear waves, which cause the oscillation of the particles of the medium at a right angle to the direction of propagation. Shear-wave speed is related to tissue stiffness and shows a great variation depending on the structure and state of tissue. Shear waves travel faster in stiff (tumor, inflamed, or fibrotic) tissues and slower in soft (normal or fatty) tissues. Recently, a quantitative estimation of tissue stiffness without using mechanical vibrations (“virtual” elastography) has been reported. This approach is based on the intravoxel incoherent motion (IVIM) contrast, which can be converted quantitatively into shear modulus [18].

#### Biological bases of elastography

Cancer alters tissue mechanical properties. Solid tumors are typically stiffer than the surrounding healthy tissue. Several tumor characteristics may explain this feature, including high cellularity, increased tumor stroma, and increased interstitial pressure [17].

#### Interpretation guidelines

The results of an elastographic exam are usually displayed by an elastogram, including an image representing the stiffnesses of the tissues superimposing on a cross-sectional slice of the anatomy. Interpretation of acquired images is based on different method of analysis [16, 17]: (1) qualitative analysis including lesion comparison to a surrounding “normal” tissue as a reference, (2) semiquantitative methods based on strain ratios between different tissues included within the selected region of interest (ROI), and (3) quantitative parameters with parametric maps.

#### Clinical value

**Fig. 2:**
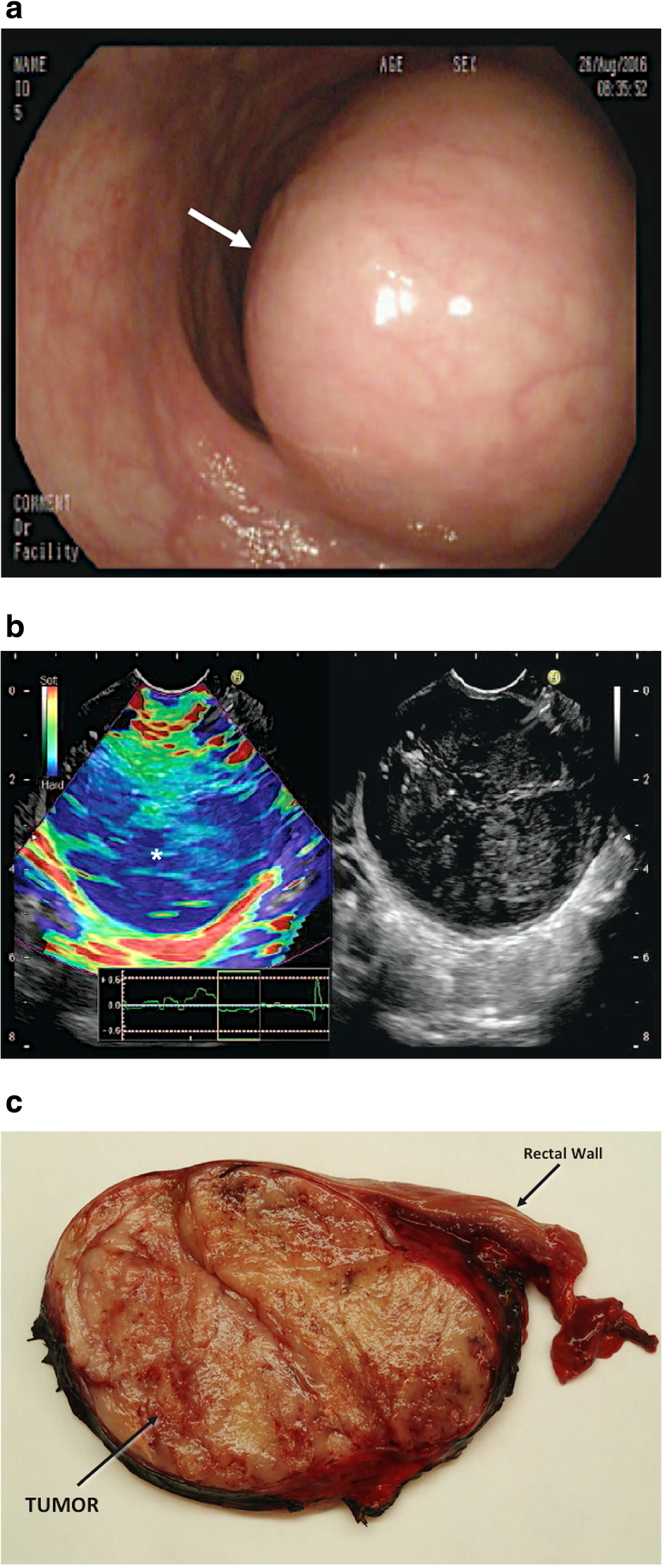
Rectal gastrointestinal stromal tumor (GIST) (white asterisk) in a 68-year-old man. **a** Optical colonoscopic image showed a rectal tumor (arrow). **b** Endorectal ultrasonographic (US) image (right) with strain elastogram (left) showed that tumor (asterisk) appeared harder (more blue) than the reference tissue on the elastogram. **c** A cut surface of the surgical specimen revealed a well-defined homogeneous aspect of the submucosal mass (GIST)

Sonoelastography enables a more accurate evaluation of the nature of superficial lesions situated in breast, thyroid, testicles, or lymph nodes (LNs). Elastography can be also performed under endoscopic ultrasound (EUS) guidance (Fig. 2). Main current clinical indications of EUS elastography are solid pancreatic lesions, submucosal gastrointestinal masses, and LNs. EUS elastography is a reliable technique for the differentiation of solid pancreatic masses with a sensitivity of 95–97% and a specificity of 67–76%, respectively [19]. In the case of the accuracy of EUS-guided elastography for the differential diagnosis of benign and malignant LNs, Xu et al. found a sensitivity of 88% and a specificity of 85% [20]. Emerging applications of EUs include prostate cancer (PCa) and rectal tumors. Elastography has been demonstrated to be superior to transrectal EUS alone in PCa, improving the specificity of prostate biopsies.

On its part, MRE has been tested in different tumor locations, including breast, prostate, liver, pancreas, or brain suggesting that this technique may improve tumor detection and characterization [17]. Preliminary studies with different therapies have showed an early decrease in tumor stiffness and viscoelasticity (with no change in tumor apparent tumor diffusion coefficient [ADC]), following treatment, suggesting that MRE may be more sensitive than DWI to early tumor response to therapy [17].

## Tumor microstructure and composition on imaging

Clinical imaging may provide information about microstructure, organization, composition, and histological features in tumors [21–26]. Different imaging techniques may be useful for this, including diffusion and relaxation-weighted MRI, magnetization transfer (MT) techniques, and spectral CT.

### Diffusion-weighted imaging

Diffusion is a fundamental imaging technique that allows a noninvasive assessment of tissue microstructure [21, 24, 25, 27–34].

#### Technical features

**Fig. 3:**
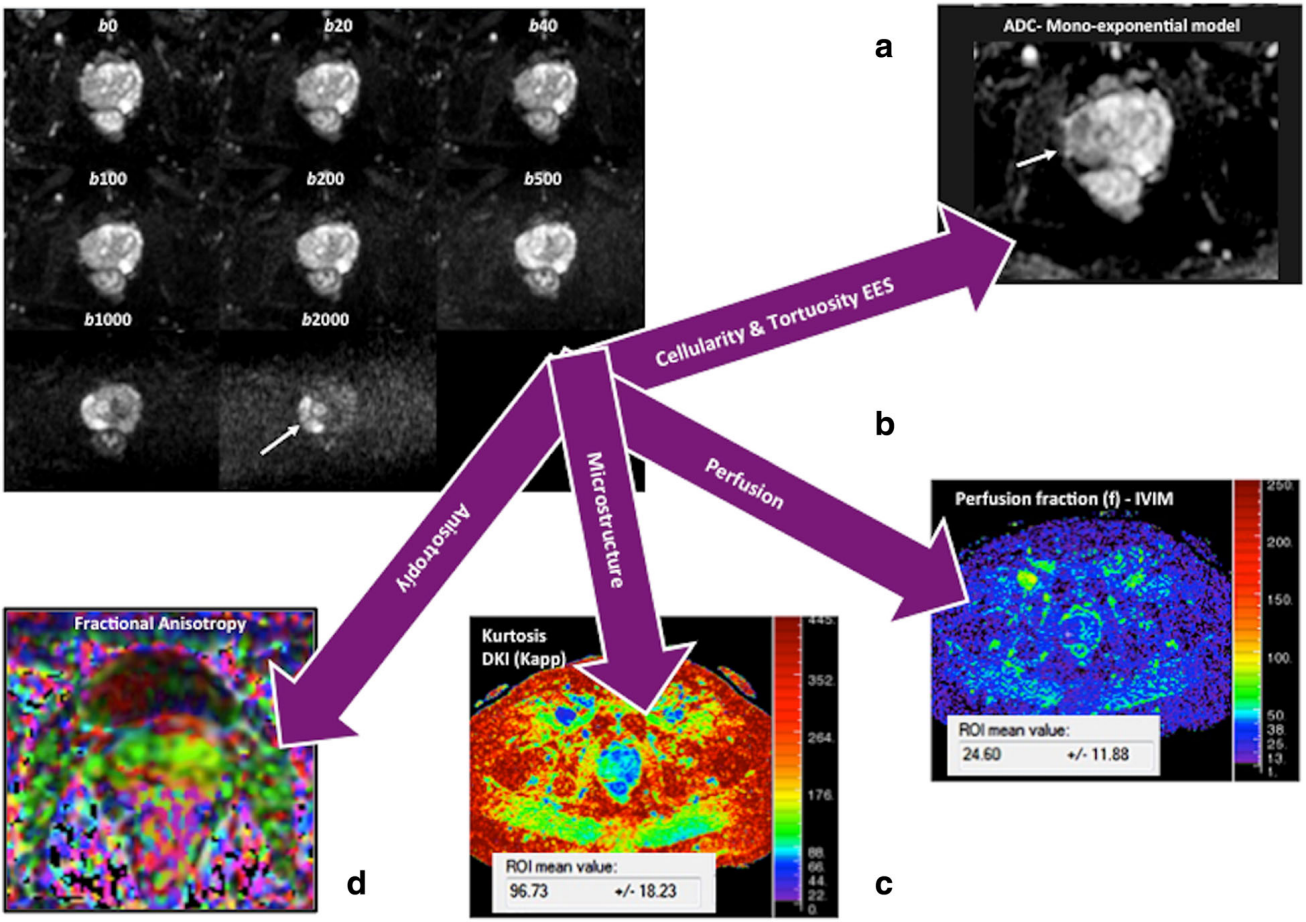
A 69-year-old man with Gleason 4 + 3 prostate cancer (white arrows). Diffusion signal evaluated using different models of analysis: mono-exponential (**a**) intravoxel incoherent exponential model (IVIM) (**b**), diffusion kurtosis imaging (DKI) (**c**), and diffusion tensor imaging (DTI) (**d**). DWI can offer multiple parameters depending on the model of analysis, including ADC, perfusion fraction (*f*), or apparent kurtosis (Kapp) with a different biological meaning. Biological and physiological correlation of parameters obtained from analysis is not entirely clear and their clinical value may depend on tumor type

**Fig. 4:**
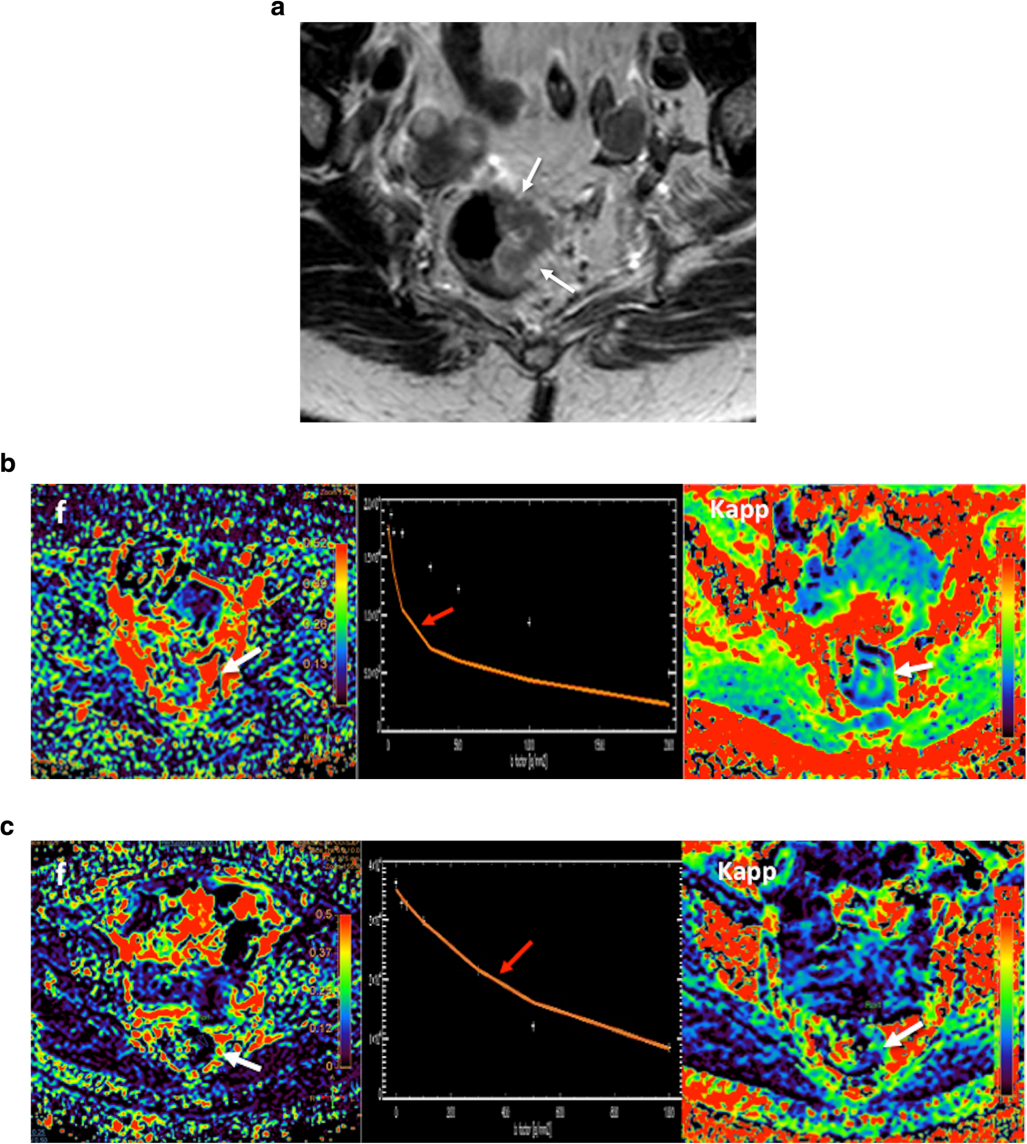
Rectal cancer in a 65-year-old woman. **a** Axial T2-weighted image demonstrated a rectal mass deeply extending to the mesorectal fat (white arrow). **b** Perfusion fraction (*f*), apparent kurtosis diffusion (Kapp), and the curve of signal intensity decay in diffusion pretherapy. Note that this curve showed a marked attenuation of signal intensity at low *b* values (red arrow) due to the influence of tumor perfusion on diffusion. **c** Following therapy, there are reduction of both f and Kapp and dissapearance of the fast attenuation of the signal intensity at low *b* values, related to tumor response with reducer perfusion on IVIM model

The extent of water molecule motion in tissues can be imaged by applying balanced gradients placed symmetrically about a fat-suppressed T2-weighted sequence. The weighting of the applied diffusion gradients is indicated by their *b* value (measured in s/mm^2^), which depends on the amplitude and duration of the gradients and the time between them. In the absence of water molecules motion, the phase shifts due to the two gradient pulses will cancel without a significant change in the measured signal intensity (SI). By comparison, in the case of moving water molecules, their signal will not be completely rephased by the second gradient, thus leading to a signal loss. Recent technological advances in diffusion-weighted imaging (DWI) acquisition have facilitated the acquisition of multiple *b* values, including high *b* values (> 1000 s/mm^2^), in clinical practice. Besides, non-Gaussian analytic models of DWI (IVIM, diffusion kurtosis imaging [DKI], stretched-exponential model [SEM]) and vascular, extracellular, and restricted diffusion for cytometry in tumors (VERDICT) were proposed to more closely reflect the distribution of physiologic and pathologic characteristics of tissues, including cellularity, microcirculation, and heterogeneity (Fig. 3). The IVIM model assumes a bi-exponential decay of signal with increased *b* values. At low *b* values (< 200 s/mm^2^), there is a deviation from the mono-exponential decay, which is due to incoherent motions of water molecules inside the microvasculature (Fig. 4) [35, 36]. On its part, DKI model analyzes the deviation of tissue diffusion from a Gaussian pattern at ultra-high *b* values (typically > 1000 s/mm^2^) [37]. SEM evaluates the effects of sub-voxel heterogeneity in diffusion. Finally, VERDICT couples DWI to a mathematical model. Prelimminary data suggest that it may allow the assessment of tumor features such as cell size, vascular volume fraction, and intra- and extracellular volume fractions [24, 25].

#### Biological bases of DWI

Diffusion measures the random (Brownian) motion of water molecules at microscopic level. However, diffusion is not free in tissues and is modified by interactions with cellular packing, intracellular organelles, membranes, and macromolecules and by macroscopic water movements (e.g., within blood vessels and glandular ducts). The use of DWI in tumor evaluation is based on the assumption that malignant tumors are usually more cellular and have a more complex extravascular-extracellular space than benign tumors/normal tissues, causing a lower attenuation of the signal in tumors with increased *b* values [21, 28–31]. As a general rule, water diffusion decrease with the increased cellularity and cell size (24b). Negative correlations between the ADC values and cellularity or proliferation have been reported in many tumor types; though, there were no clear correlations in others [32, 33]. It must be also considered that molecular mobility is anisotropic, not equal for all directions. Based on this, diffusion tensor imaging (DTI) may describe the magnitude, degree, and orientation of diffusion anisotropy and may estimate the tissular microstructure. Characterizing the non-Gaussian diffusion MRI signal behavior may also provide valuable information on tissue structure and function. IVIM model allows the separation of pure diffusion characteristics from pseudodiffusion and perfusion features. IVIM-derived parameters include the pure molecular diffusion coefficient (*D*), the perfusion-related diffusion coefficient (D*), the perfusion fraction (*f*), and the relative perfusion (fD*) (Fig. 4). The biological meaning of these parameters is not entirely clear. However, the *f* values have been suggested to be related to the blood volume (BV) and have been significantly correlated with the percentage of arterial enhancement in different tumor types [34–36]. On its part, D* and fD* may hold information on blood speed and on the quantity of blood flowing through a unit tissue per unit time, respectively [34]. Most studies have also found a fair to good correlation between *f* and histological surrogate markers of angiogenesis, such as the microvessel density (MVD), and parameters derived from perfusion imaging techniques [34–36]. DKI provides an additional model of diffusion analysis at higher *b* values (> 1000 s/mm^2^), in which the signal contribution of water from the extracellular space is substantially reduced, making the diffusion measurement more sensitive to the motion of water in the intracellular compartment. However, in the case of DKI- and SEM-derived metrics (e.g., apparent kurtosis [*K*_app_] or the stretching parameter [α]), these parameters do not have a simple biological interpretation; although presumably may reflect tissular heterogeneity (Fig. 4) [37].

#### Interpretation guidelines

- Qualitative evaluation

**Fig. 5:**
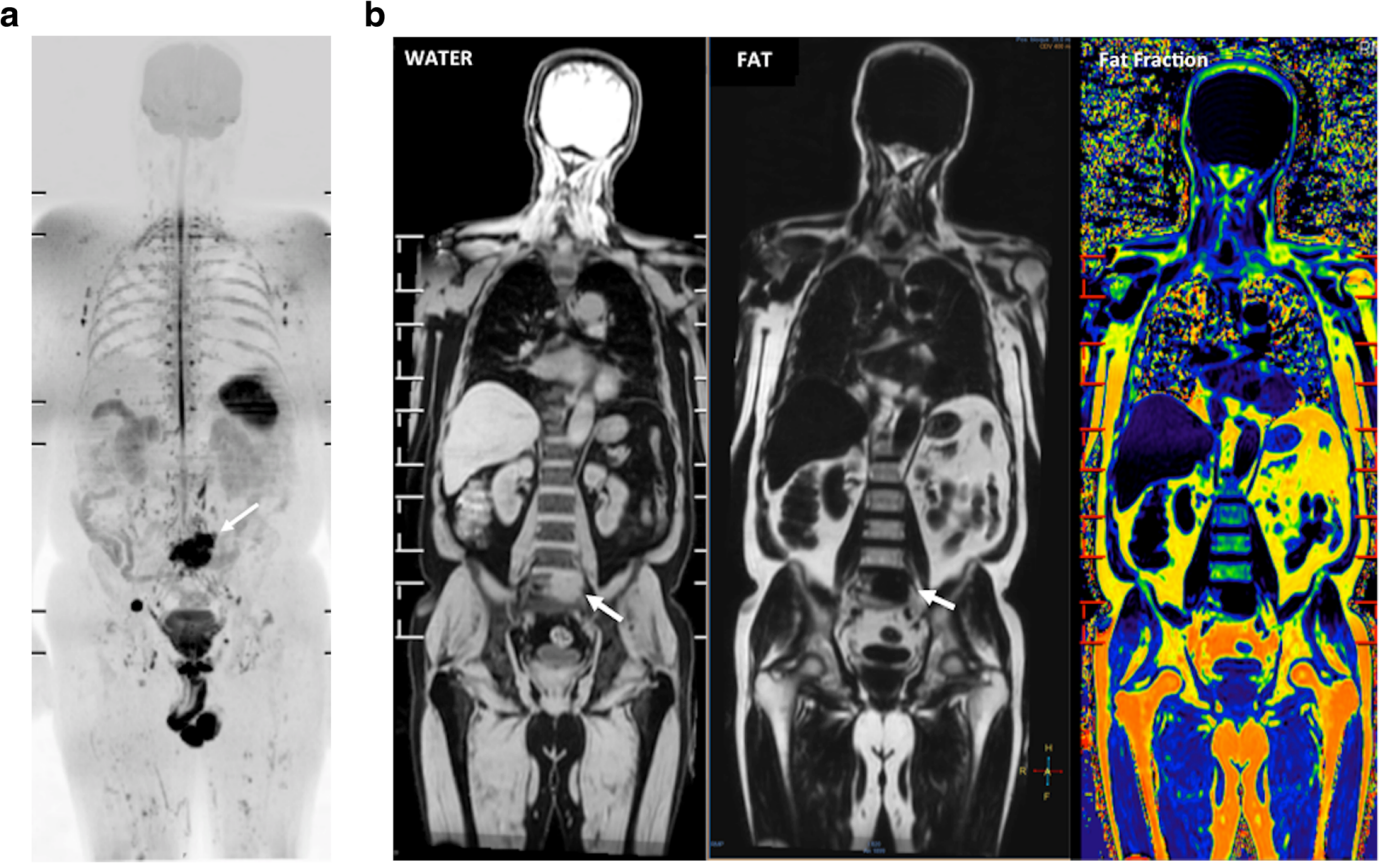
A 68-year-old man with lung adenocarcinoma. **a** The whole-body diffusion weighted MR image (*b* = 900 s/mm^2^) depicted a metastatic deposit in the sacrum (white arrow). **b** Coronal reformatted T1W images obtained using the Dixon technique water only (WATER) (left), fat only (FAT) (middle), and fat-fraction in colored scale (right) evidenced not fat content and increased water in the metastatic lesion (white arrow)

The diffusion signal alterations serve as an excellent qualitative tool, providing an additional contrast mechanism to supplement routine conventional sequences. Areas retaining high SI on high *b* values images suggest highly cellular tissues, such as tumors [28–31] (Fig. 5). This feature may be a useful clinical tool. A recent meta-analysis reported that visual assessment of tumor diffusion might be more accurate than ROI measurements of ADC for PCa detection [38]. However, high signal intensities on DWI are not always reliable indicators of increased cellularity, and high *b* values images, ADC maps, and co-registered anatomical images should always be evaluated together (Table 1).

- Quantitative evaluation

**Table 1:** Interpretation of tumor diffusion-weighted images

Signal intensity on high b-value images (b800–b1000)	Relative value on apparent diffusion coefficient (ADC) maps	Signal intensity on T2-weighted images	Interpretation
High	Low	Intermediate	Generally, high cellularity tumor Coagulative necrosis Abscess Rarely high protein content
High	High	High	T2-*shine through* (often proteinaceous fluid)
Low	Low	Low	Fibrous tissue with low water content +/- viable tumor
Low	High	High	Fluid Liquefactive necrosis Lower cellularity/grade tumor Glandular tissue
Low	High	High	Vasogenic edema (T2-*wash out*)
Low	Low	Variable	Hemorrhagic content (T2-*black out*)

The ADC has served as a quantitative biomarker for the evaluation of diffusion in clinical practice. Calculation of an ADC value is typically performed utilizing a mono-exponential fit of the diffusion signal at different *b* values. There is a linear decay of the natural logarithmic diffusion SI as the *b* value is increased. The slope of this line is the ADC value (units 10^−3^ mm^2^/s). Malignant lesions usually have lower ADC values compared to surrounding normal tissue, edema, and benign tumors [28–31]. Unfortunately, there is considerable disparity in the published ADC values across different anatomies, vendors, or technical parameters of the acquisition causing that there are no unique cut-off ADC values that distinguish cancer from normal tissues. Besides, both well-differentiated tumors and necrotic poorly differentiated tumors may show high ADC values and some normal tissues (including endometrium, bowel mucosa, testes, and normal LNs and nerves) may show increased SI on high *b* value [28–31]. Finally, non-Gaussian models yield several quantitative parameters that may improve diffusion assessment. Malignant lesions usually present high values of *f* and *K*_app_ and lower values of α [27, 34, 37].

#### Clinical value

**Fig. 6:**
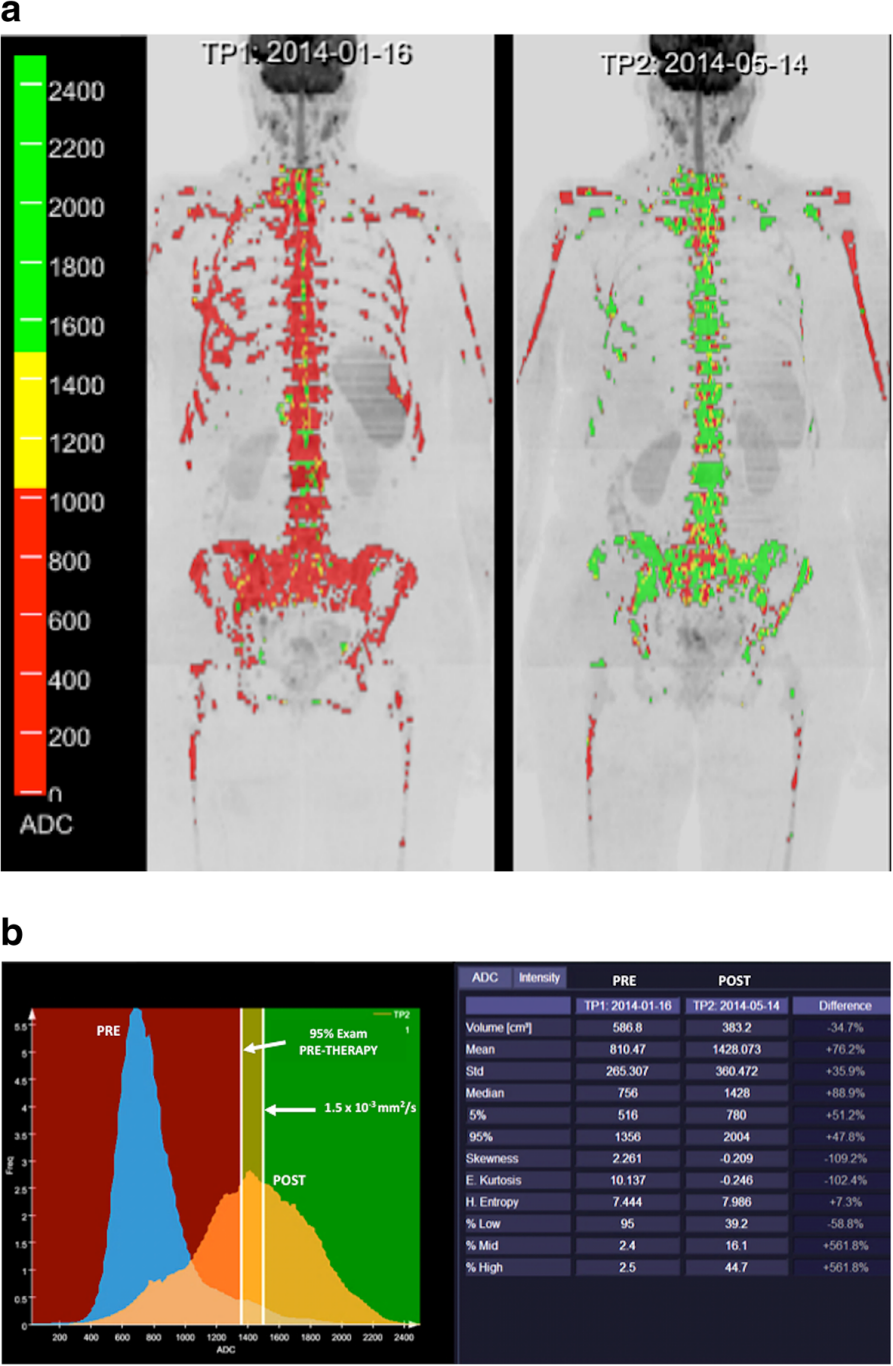
A 52-year-old woman with metastatic breast cancer treated with chemotherapy (**a**). Whole body diffusion-weighted inverted gray-scale maximun intensity projection (MIP) of *b* = 900 s/mm^2^ images superimposing the ADC values associated with each voxel in color scale before (left) and after one cycle of therapy (right). Red colored voxels represent untreated disease or those with no-detectable response. Thus, yellow voxels represent regions “likely” to be responding. Green colored voxels have ADC values ≥ 1500 μm^2^/s representing voxels that are “highly likely” to be responding with tumor cell kill. Tumor evaluation showed a great change in ADC values (predominantly green voxels with ADC values ≥ 1500 μm^2^/s) indicating tumor necrosis. **b** A detailed ADC analysis of histogram metrics evidenced a reduced tumor volume as well as improvement in several histogram metrics (mean ADC, kurtosis, etc.) that have changed significantly during treatment due to extensive necrosis (compare to Fig. 7)

**Fig. 7:**
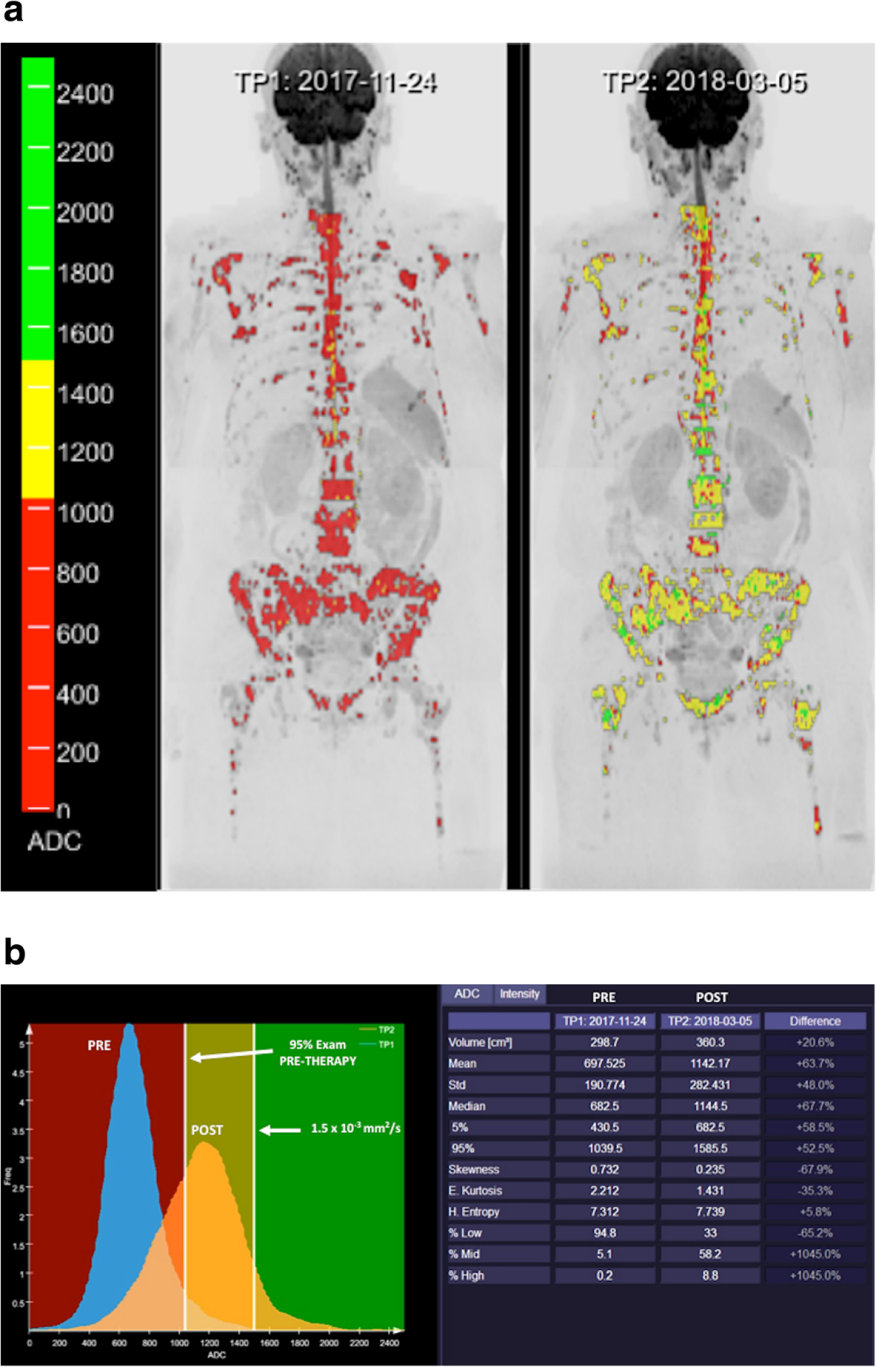
A 63-year-old woman with metastatic breast cancer treated with an anti-HER2 agent and hormonotherapy. **a** Whole body diffusion-weighted inverted gray-scale maximun intensity projection (MIP) of *b* = 900 s/mm^2^ images superimposing the ADC values associated with each voxel in color scale before (left) and after one cycle of therapy (right). Red colored voxels represent untreated disease or those with no-detectable response. The yellow voxels lie between the 95th centile value of the pre-treatment histogram and 1500 μm^2^/s. Thus, yellow voxels represent regions “likely” to be responding. Green colored voxels have ADC values ≥ 1500 μm^2^/s representing voxels that are “highly likely” to be responding with tumor cell kill. In this case, response assessment demonstrated a discrete change in ADC values (predominantly yellow voxels). **b** The detailed ADC analysis of histogram metrics evidenced an increased tumor volume with moderate changes in several histogram metrics (mean ADC, kurtosis, etc.) suggesting an apoptotic effect of targeted therapies (compare to Fig. 6)

DWI has shown clinical value for tumor detection, diagnosis and grading, staging, prognosis, therapy monitoring, detection of tumor relapsing, and assessment of patient’s outcome [27, 31, 34, 39–44]. Malignant tumors are generally more cellular than benign tissues and show lower ADC values. However, false positive results may occur with abscesses and infective processes and false negatives may happen with cystic, necrotic lesions and in well-differentiated neoplasms (particularly adenocarcinomas) [27–30]. DWI has also demonstrated to be an effective tool for evaluating tumor response to therapies [40–44]. Successful treatment is usually reflected by increases in ADC values. However, it depends on the mechanism of action of therapy given. Transient decreases in ADC can also be observed in patients treated with anti-VEGF therapies in brain tumors [43, 44]. This finding appears to be related to cellular swelling and reductions in tumor BF, extracellular space, and vasogenic edema. Accurate imaging response evaluations of oncologic patients are sometimes notoriously difficult with conventional imaging techniques, especially in the case of bone lesions. In this setting, whole-body (WB)-DWI MRI has shown clinical value for the assessment of therapeutic response in patients with metastatic bone disease, multiple myeloma, and lymphoma [41, 43–54] (Figs. 6 and 7). Finally, regarding the prognostic/predictive value of ADC in tumor assessment, lower ADC values are usually associated to patients with poor outcome; although are correlated to a favorable response to most treatment options.

IVIM represents an alternative to perfusion contrast-enhanced techniques without the use of contrast agents in oncologic imaging. IVIM-metrics (mainly *f*) have demonstrated to be useful for tumor diagnosis and characterization in liver, pancreatic, breast, H&N, brain, renal, cervical, and rectal tumors [35, 55]. IVIM has also shown promising results for monitoring therapy response (especially antiangiogenic drugs or vascular targeting agents) in clinical practice [21, 35, 55].

Clinical applications of kurtosis have been mainly focused on prostate, breast, H&N, lung, renal, and brain tumors. Published data suggests that DKI may provide additional information and may improve tumor diagnosis and characterization compared with conventional diffusion parameters in these tumor types [37]. Up to now, SEM has been used in a limited way in the evaluation of tumors. Preliminary data of the use of this technique have been published in prostate, breast, cervical, rectal, ovarian, and H&N cancers and gliomas [37].

### Tissular composition and imaging

Imaging (specially MRI) may allow the depiction of tumor composition. Several tumor types show characteristic imaging findings. For instance, melanin (a paramagnetic substance) shortens the T1 relaxation time, making melanoma appear hyperintense on T1-weighted images. MRI is a useful tool for the assessment of fat content, T1, T2, or T2* mapping, or MT. MRI fat quantification allows monitoring bone marrow (BM) composition in oncologic patients and BM changes that result from therapy. BM has variable composition and vascularization (Fig. 5). Yellow BM is primarily composed of fat (> 80%), while red normal red BM or bone tumor infiltration usually show and increased percentage of water (30–40% water in normal red BM). These features may be useful in the evaluation of patients with metastatic bone disease for diagnosis or therapy response assessment [43, 50, 53, 56–58]. Besides, each biological tissue has a T1 and T2, or T2* relaxation times based on its cellular and interstitial components. T1 mapping can detect important tissular characteristics such as excess of water (e.g., edema), protein deposition, and the presence of other T1-altering substances, such as lipid or iron (hemorrhage, siderosis). Quantitative T1 mapping requires a series of images using different inversion times to derive a T1 recovery curve resulting in a map that describes the relaxation value on a pixel-by-pixel basis, expressed in milliseconds. In oncology, studies about the use of T1 values in tumors evidenced that this parameter was greater than in normal tissues due to an increased extracellular space. On the contrary, low tumor T1 values have been correlated to increased necrosis, low water content, and high levels of proteins [59]. Native T1 mapping could also represent an in vivo biomarker for the differentiation of tumor grade [60]. Significant changes in T1 were shown in several pre-clinical models in response to therapy [59, 61]. The use of T2 mapping in oncology has been mainly focused on PCa. Sabouri et al. have demonstrated the feasibility of T2-derived parameters (e.g., luminal water fraction) in the detection and grading of cancer [62]. T2 values were significantly lower in Gleason ≥ 7 than in Gleason ≤ 6 cancers in peripheral zone, but this parameter will not be sufficient by itself to adequately characterize focal abnormalities. An explanation of this feature may be the effect of normal prostate tissue interdigitated with malignant glands, which may reduce contrast between tumor and normal prostatic tissue [63]. Finally, quantitative T2* mapping seems also to be a method that may be potentially useful for characterizing malignant tumors. In this setting, T2* mapping showed greater diagnostic accuracy than ADC mapping in the characterization of intermediate- and high-grade PCa, although it also demonstrated a limited value in the characterization of low-grade PCa [64]. In the case of breast cancer, the T2* value is significantly longer in invasive cancers compared with ductal carcinoma in situ and in high-grade tumors [65]. Unfortunately, a reliable diagnosis of malignancy cannot be made on the basis of a quantitative evaluation of T1, T2, or proton density indexes.

**Fig. 8:**
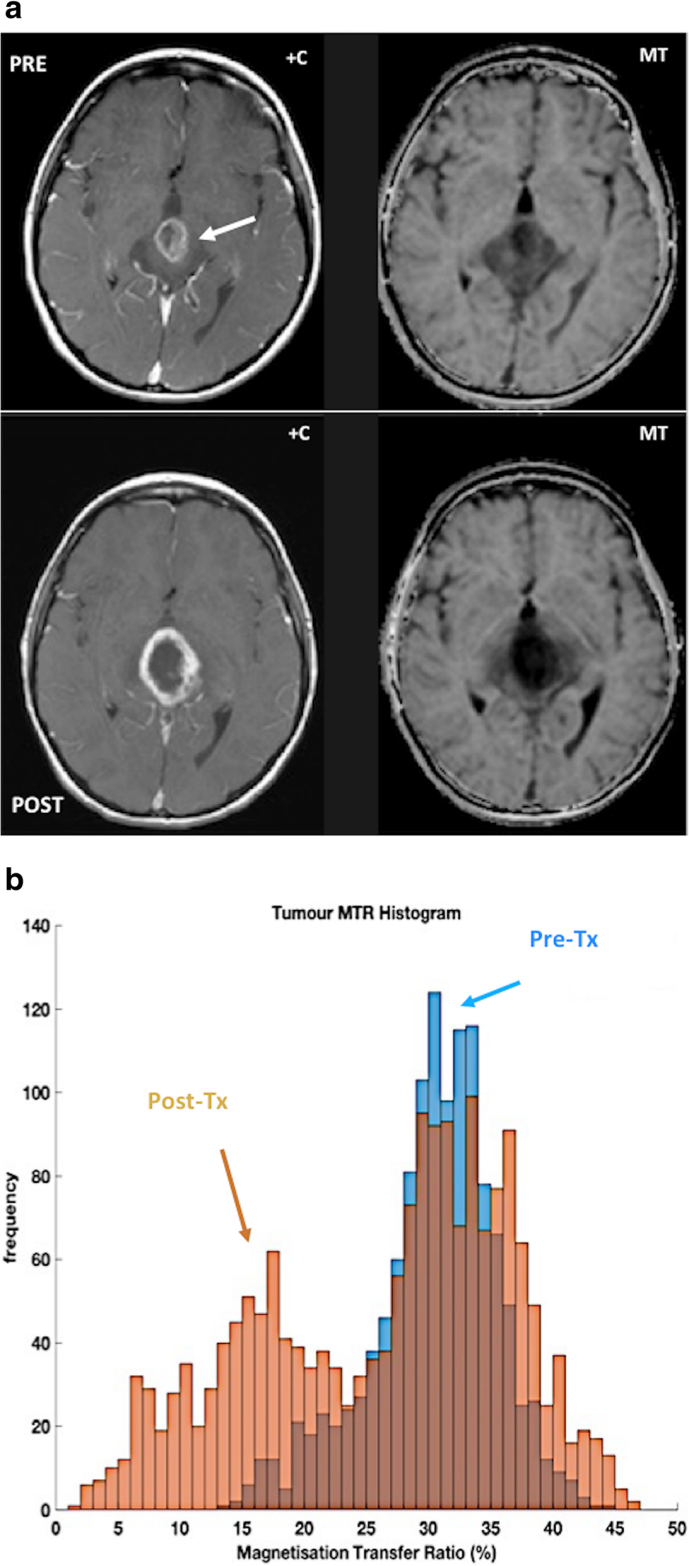
An 8-year-old patient with high-grade tectal plate glioma (white arrow). **a** Post-contrast T1-weighted (+C) and magnetization transfer (MT) images were obtained before (top row) and after (bottom row) chemoradiotherapy; and (**b**) the corresponding MT ratio histograms of lesion are as shown. The magnetization transfer acquisition comprised a set of four geometry-matched 3D-GRE scans: two flip angles (4 and 24°), with/without MT pulse (1.5 kHz offset) on a 1.5 T MR system. Note the reduction in the MT ratio (histogram in **b**) associated with response to treatment, accompanied by slight increase in size and enhancement of the tumor (**a**). [Images courtesy of Dr. Neil P. Jerome, Norwegian University of Science and Technology]

MT imaging and chemical exchange saturation transfer (CEST) can evaluate the presence of molecules other than water. MT may be useful for evaluating tissues with significant water-macromolecule interactions. This technique has showed promising results as a possible tool for tumor assessment in prostate, testicle, rectal, breast, and brain tumors (Fig. 8) [66]. On its part, CEST is just one type of MT. CEST can detect low concentrations of molecules through the presence of ^1^H protons that are exchangeable with those of water causing a decrease of signal intensity. Amide proton transfer (APT) imaging is the most widely used CEST technique. APT imaging for cancer assessment has been mainly focused on the brain, but higher APT values have been found in malignant tumors compared with those in normal tissues and benign lesions in brain, breast, prostate, chest, and H&N. APT values also varied between malignant groups and tumor grades [67, 68].

Spectral CT also enables material identification (calcium, fat, etc.) and quantification, providing increased tumor detection and characterization [69]. In this setting, for example, Kosmala et al. reported that material-specific image processing may facilitate the identification of BM tumor infiltration [70].

## Tumor microenvironment

The biology of tumors can no longer be understood simply by evaluating tumor cells but instead must include the contributions of the tumor microenvironment (TME) to tumorigenesis. TME is a complex, heterogeneous, and dominant component of solid tumors. TME includes the acellular component (named the extracellular matrix, ECM) and different co-opted cell types, including cancer-associated fibroblasts, mesenchymal cells, and immune infiltrate. The non-malignant cells of the TME can comprise > 50% of the mass of tumor burden. TME plays critical roles in both promoting the malignant progression of solid tumors and modifying the response of solid tumor cells to therapy. Pathological TME usually shows insufficient oxygenation (hypoxia) and acidosis. Multiple imaging modalities have been employed to evaluate the TME, but most of them are still at the pre-clinical phase of testing [71–73].

**Fig. 9:**
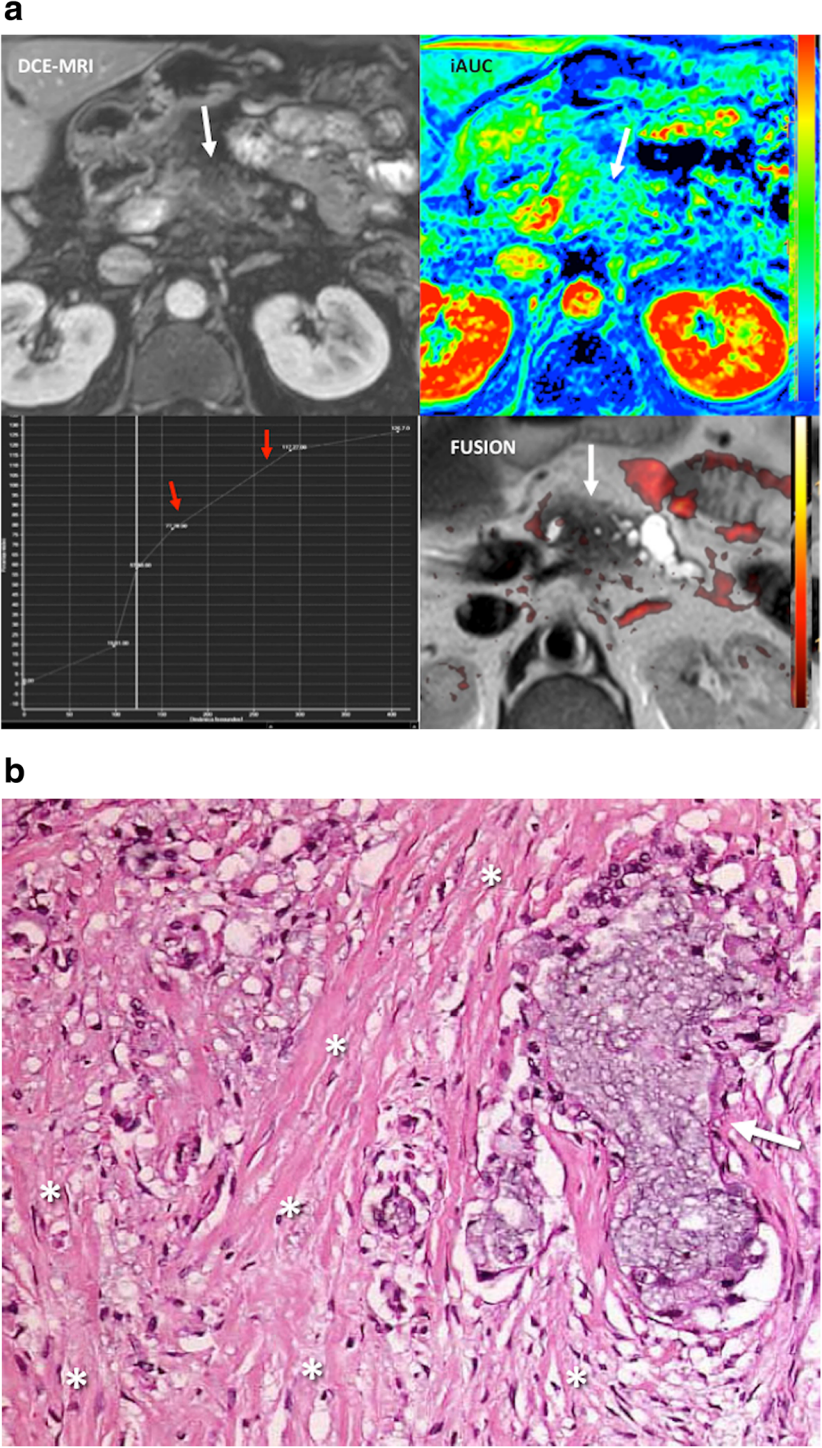
Desmoplastic reaction in a 51-year-old patient with pancreatic adenocarcinoma. **a** Imaging evaluation including an axial fat-suppresed contrast-enhanced T1-weighted dynamic MR image (DCE-MRI, top, left), a time/signal intensity curve analysis (botton, left), a parametric map corresponding to the initial area under the curve (iAUC), and a fusion of axial T2-weighted image fused with a superimposed color-coded map derived from high *b* value DWI (FUSION) clearly showed a hypoperfunded mass with a pattern of progressive enhancement (curve type 1, red arrows) and no restricted diffusion in the tumor in the fusion image. These findings were secondary to the predominance of fibrosis within the lesion. **b** Histological analysis (H&E, × 20) confirmed an abundant tumor desmoplastic reaction (asterisks) with clusters of tumor cells (arrow)

### Imaging of tumor stroma

Apart of the study of tumor vascularization, imaging evaluation of tumor stroma has been scarce. The stroma can make up a significant proportion of a tumor volume, and differs from normal stroma, showing a high number of fibroblasts, deposition of type I collagen and fibrin, and a marked infiltration of inflammatory cells. Stroma may deeply influence imaging findings of tumors (Fig. 9). For instance, in the case of pancreatic cancer, neoplastic cells may only represent as little as 10% of tumor volume in pancreatic adenocarcinoma, associated to the presence of abundant desmoplastic stroma. Although these tumors usually show increased angiogenesis on histological analysis, tumor imaging findings are deeply modified by stromal fibrosis, a feature which explains that pancreatic adenocarcinomas usually show a diminished enhancement in the early phase of dynamic contrast-enhanced imaging techniques and gradual enhancement in the late phase (Fig. 9).

**Fig. 10:**
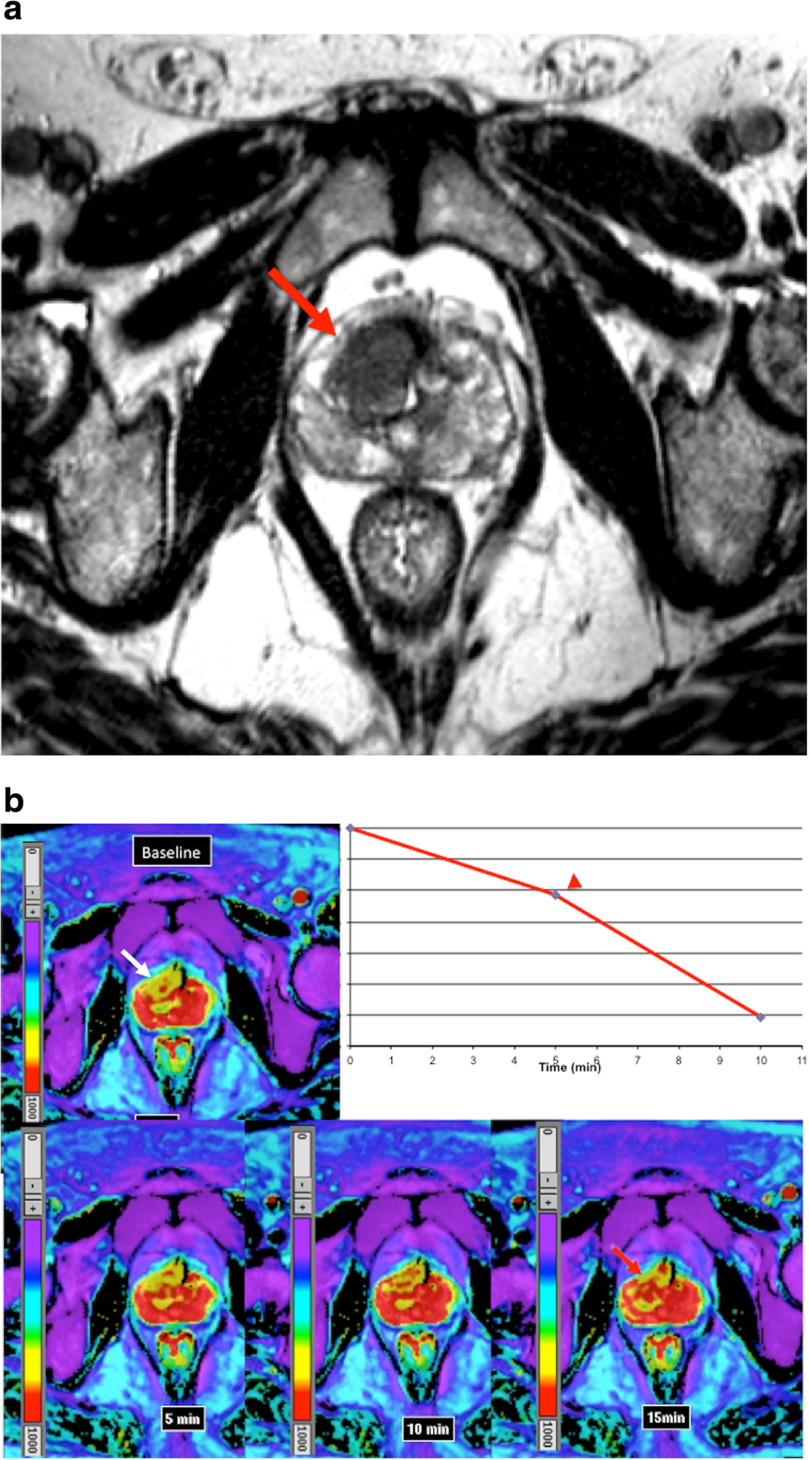
BOLD sequence and tumor oxygenation. **a** Axial T2-weighted image in a 63-year-old man evidenced a poor defined hypointense mass in the anterior part of the transitional gland corresponding to prostate cancer (white arrow). **b** BOLD exam acquired in the axial plane at baseline and after evidenced that in baseline conditions the tumor (white arrow) showed low signal in the T2* map (high R2* values), which is related to a lower oxygenation compared to the rest of the prostate. BOLD images after 95% oxygen breathing at 5, 10, and 15 m evidenced that the signal increased witihin the tumor (red arrow in the acquisition at 15 min) with an inverted ∆R2* time-intensity curve (red arrowhead). These features evidence the presence of radiosensitive areas within the tumor with increasing pass of oxygen from blood to the tissue. The concentration of deoxyHb increases with rising oxygen consumption, leading to a decreasing T2* relaxation time of the surrounding tissue

### Imaging of tumor-infiltrating immune cells

Interactions between tumor cells and immune cells are involved on the initiation, progression, therapy-resistance, and prognosis of cancer. Besides, immunotherapy has successfully been introduced in the clinic for many cancer types (especially in cancer types with high mutation rates). To date, there is a limited experience with the evaluation of tumor-infiltrating immune cells and responses to immunotherapy in clinical practice and there is a lack of imaging tools to measure the behavior of immune cell populations, which is hampering the optimization and individualization of immunotherapy [74].

### Imaging of oxygenation and hypoxia in cancer

Tumor hypoxia leads to treatment resistance, enhanced tumor progression, and has a negative impact on patient prognosis and survival in cancer. Hypoxia changes the pattern of gene expression resulting in a more aggressive tumor phenotype [75]. Oxygen-enhanced MRI, including blood oxygenation level dependent (BOLD) and tissue oxygen level dependent (TOLD) techniques, and PET imaging based on mitroimidazole analogues and complexes of copper with diacetyl-bis(N4-methylthiosemicarbazone) (ATSM) provide a noninvasive assessing of tumor oxygenation [75–77].

#### Biological bases of tumor hypoxia

Uncontrolled cell proliferation and the inability to form normal blood vessels results in impaired blood supply and low oxygen tension within tumors. Hypoxia activates adaptive cellular responses and genomic instability that contribute to tumor progression and is associated with poor prognosis and resistance to different therapies, potentially contributing to poor patient survival. Two main types of tumor hypoxia are recognized [76, 77]: (1) acute (perfusion-related) hypoxia resulting from inadequate BF in tumors due to structural and functional abnormalities of tumor vasculature and (2) chronic hypoxia, which is the most relevant type in oncology. Two fundamental mechanisms of chronic hypoxia can be differentiated: diffusion-limited hypoxia (caused by increased oxygen diffusion distances due to tumor growth) and hypoxia due to a compromised perfusion due to inefficient and leaky microvessels.

#### Technical features

- Oxygen-enhanced MRI

BOLD-MRI contrast derives from variations in the magnetic susceptibility of blood due to changes in the concentration of deoxyhemoglobin. Deoxyhemoglobin increases the transverse relaxation rate (R2*) of water in blood and surrounding tissues. This change in magnetic susceptibility produces local magnetic fields around blood vessels, changing signal intensity on MR images (Fig. 10). BOLD provides an indication of tumor blood oxygenation, but is also sensitive to vessel density, blood flow hematocrit, and pH [77]. On its part, TOLD MRI is based on T1-weighted contrast and the measured R1 (=1/T1) is also sensitive to tissue oxygenation.

- Radiotracers

**Fig. 11:**
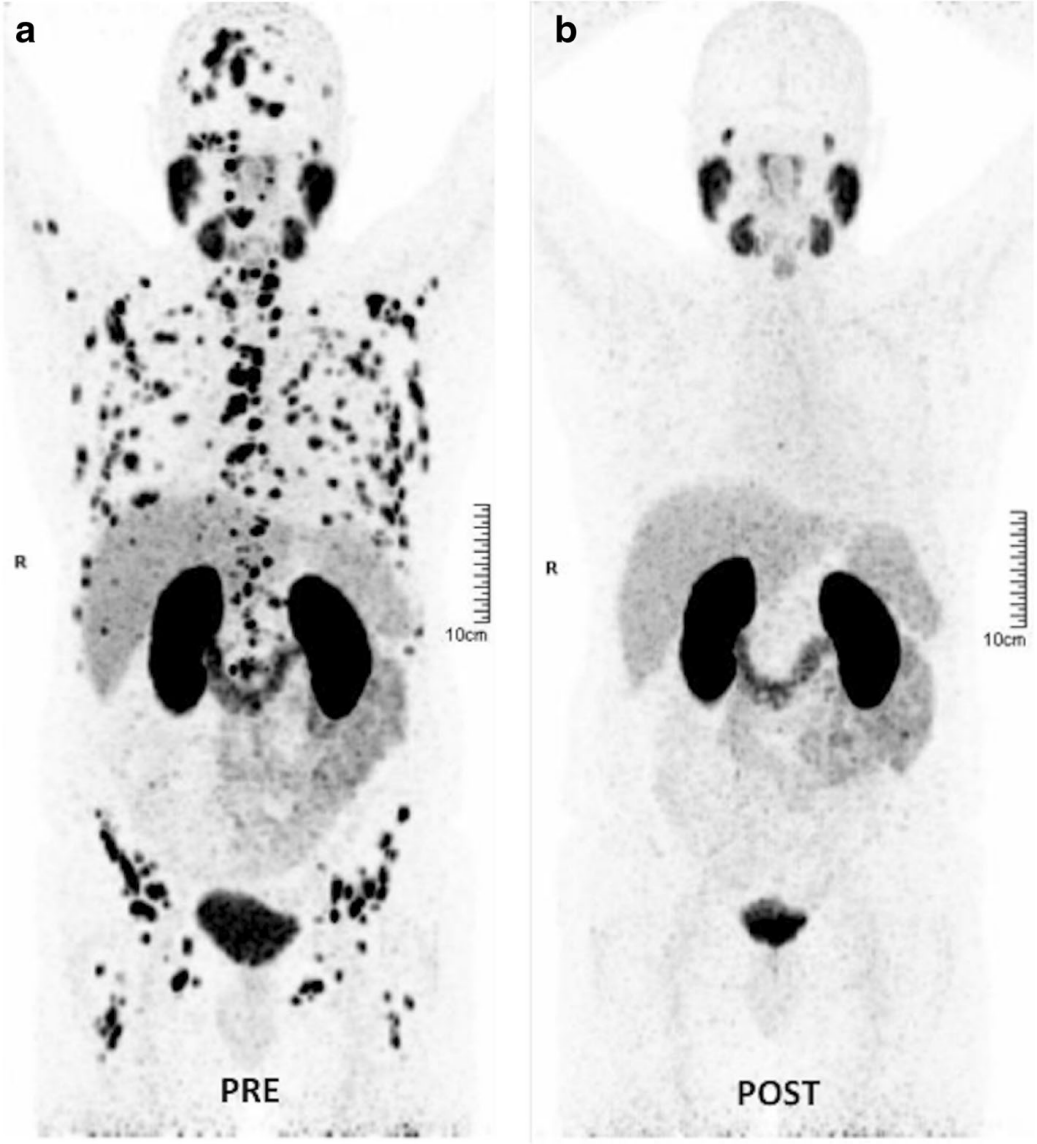
Theranostic in oncology with PSMA. ^177^Lu-PSMA radioligand therapy in a 67-year-old man with metastatic castration-resistant prostate cancer. **a** Pretherapy. PET image evidenced a difuse metastatic involvement. PSA value 50 ng/ml. **b** 4 months following the treatment with ^177^Lu-PSMA radioligand therapy (8000 MBq), PET showed a complete metabolic response. PSA value 0 ng/ml

^18^F-fluoromisonidazole (^18^F-FMISO) is the main mitroimidazole analogue and constitutes the most extensively clinically studied PET hypoxia biomarker. FMISO enters cells under hypoxic conditions and becomes trapped at rates that are inversely proportional to the local pO_2_. ^18^F-FAZA is another PET imaging tracer of tumor hypoxia that offers the advantage of higher tumor-to-reference tissue ratios in comparison with ^18^F-FMISO.

Combinations of cooper with ATSM ligands, such as ^64^Cu-ATSM, also appear to be promising radiotracers for delineating the extent of hypoxia within tumors with PET. Under hypoxic conditions, the unstable cooper-ATSM complex may dissociate, causing its intracellular trapping [78].

#### Clinical value

BOLD images do not measure tissue pO_2_ directly and are more likely to reflect acute than chronic tissue hypoxia. Moreover, it is necessary to know the distribution of blood volume (BV) in tissue in order to be able to correctly interpret R2* images, thereby permitting inference of oxygenation status. However, a significant link was found between R2* and pimonidazole histology (a marker of hypoxia) in patients with PCa [79]. The results showed that the sensitivity of R2* in depicting tumor hypoxia was high (88%), but its specificity was low (36%). Concerning the role of oxygen-enhanced MRI in clinical practice, several published studies have shown a value for assessing tumor oxygenation and predict radiation response [80].

On its part, PET imaging of hypoxia has demonstrated clinical value in many different tumor types facilitating the identification of hypoxic tumor areas, predicting prognosis and response to treatment, improving radiotherapy planning, and allowing the development of hypoxia therapeutics by measuring response to hypoxia-modifying treatments.

### Imaging of tumor pH (acidosis)

Glycolytic metabolism and the hypoxic microenvironment lead to extracellular acidosis in solid tumors. Major approaches for pH imaging include MRS, MT methods, and pH-dependent relaxation agents, although with a limited clinical use [81]. Published preclinical data have evidenced the presence of an acidic extracellular pH and alkaline intracellular pH in tumors relative to normal tissues [76, 82, 83].

### Imaging the expression of specific molecular characteristics

Tumor cells may overexpress some specific receptors, antigens, and proteins. Specific markers such as the prostate-specific membrane antigen (PSMA) (Fig. 11), the epidermal growth factor receptors (EGFR), the human epidermal growth factor receptor-2 (HER-2), the androgen receptor, the somatostatin receptors (SSTR), the C-X-C motif chemokine receptor 4 (CXCR4), and bombesin receptors represent excellent examples of targets for cancer imaging and therapy in different tumor types [84] (Table 2).

**Table 2:** Radiotracers in the evaluation of tumor characteristics

Radiotracer	Biological Correlation	Tumor Type	Main indications
FDG	Energetic glycolytic metabolism	Many tumor types	Diagnosis, staging, response, prognostic value, relapsing tumor
Choline radiotracers	Cellular membrane turnover and phosphatidylcholine metabolism	Prostate Bladder, Brain	Diagnosis, staging, relapsing tumor
Methionine	Amino acid transport and protein synthesis	Brain and head and neck	Diagnosis, grading, response, prognostic value, relapsing tumor
Acetate	Lipid synthesis and energetic metabolism	Prostate Hepatocellular carcinoma	Diagnosis, staging, relapsing tumor
DOPA	Dopamine uptake and metabolism	Neuroendocrine tumors	Diagnosis, staging, relapsing tumor
FLT	Cellular proliferation and tyrosine kinases-1 activity	Lung, Lymphoma, Colorectal. Gastric and Pancreas	Diagnosis and tumor response
Sigma-2 (σ2) Receptor	σ2 receptors are expressed in proliferating tumor cells		Diagnosis and treatment evaluation
Integrin targeted Imaging agents (RGD) and VEGFR targeted Imaging agents	Angiogenesis		Preclinical use
Annexin V	Tumor Apoptosis		Preclinical use
Nitroimidazoles (FAZA, FMISO) Cu-ATSM	Tumor hypoxia		Preclinical use
Radiotracers Specific Tumor Types			
Radiotracers Targeting Specific Tumor Markers	EGFR expression PSMA expression Chemokine receptor type 4 (CXCR4) DOTA-peptides (Somatostatin receptors) Bombesin receptors	Lung, Colorectal Prostate Breast and head and neck cancer metastasis Neuroendocrine tumors Prostate, breast, small cell lung cancer, GIST	Preclinical use Relapsing tumor. Staging and tumor response Preclinical use Diagnosis, staging, relapsing tumor, response assessment
Non-Tumoral metabolic pathways			
NaF	Bone metabolism (non-specific tumor tracer)	Bone metastases	Diagnosis and staging

*64CuATSM* diacetyl-bis(N4-methylthiosemi-carbazone) copper(II)), *EGFR* epidermal growth factor receptor, *FAZA* fluoroazomycin arabinoside, *FDG* fluorodeoxyglucose, *FLT* fluorothymidine, *FMISO* fluoromisonidazole, *GIST* gastrointestinal stromal tumors, *NaF* sodium fluoride, *PSMA* prostate-specific membrane antigen, *VEGFR* vascular endothelial growth factor receptor

#### Clinical value

An important characteristic of neuroendocrine tumors (NETs) is the expression of SSTR on their cell membranes [85]. The SSTR2, SSTR3, and SSTR5 SSTR subtypes are often overexpressed on NETs in almost 90% of cases. The determination of the status of somatostatin receptor expression is needed to select patients for peptide receptor radionuclide therapy. For a long time, ^111^In-octreotide (Octreoscan) has been considered the reference for imaging NETs. However, at present, ^68^Ga-DOTA-somatostatin analogues represent the “new gold standard” for imaging NETs, offering high sensitivity and specificity and providing important diagnostic, therapeutic, and prognostic data. On its part, EGFR overexpression has been shown to correlate with aggressiveness of tumors and poor survival of patients in many tumor types. Imaging evaluation of EGFR expression (e.g., cetuximab DOTA-labeled PET imaging) may facilitate patient selection for HER therapy and monitor treatment response [86]. The PSMA is highly expressed on most PCa, although 5–10% of primary PCa or PCa metastatic deposits have negative PSMA results on PET. PSMA expression is usually increased in advanced stages, including metastatic castrate-resistant PCa. Unfortunately, high PSMA expression has also been described in other tumor types (colon, kidney, breast, and bladder cancer), in benign lesions (e.g., schwannomas, thyroid adenomas), and normal tissues, which may cause potential imaging pitfalls [87]. PSMA-based PET imaging may have considerable influence on the management of primary (intermediate- and high-risk) PCa patients as well as early recurrent disease [88]. Besides, PSMA may also represent a possible target for therapy in advanced PCa (Fig. 11). Finally, CXCR4 plays a pivotal role in tumor development and metastasis and it is overexpressed in many solid and hematologic cancers. The CXCR4 receptor also represents a promising target for imaging and radionuclide therapy [89].

## Imaging main tumor hallmarks

The hallmarks of cancer (biological characteristics acquired during the initiation and progression of tumors) represent key features of tumors. Imaging techniques have unique potential to comprehend most of these tumor-related characteristics, including metabolic reprogramming, sustaining proliferative signaling and evading growth suppressors, resisting cell death and apoptosis, and inducing angiogenesis [1, 2].

### Tumor metabolic reprogramming

Changes in cell metabolism can contribute to malignant transformation and tumor progression. Tumor metabolic phenotypes influence tumor prognosis and treatment and can be exploited to image tumors [90]. Metabolic alterations in cancer cells include the increased generation of energy and biosynthetic intermediates needed for cell growth and proliferation. Molecular imaging techniques, which include MRSI, PET, and SPECT imaging, can assess the altered metabolic profiles of tumors [91].

### Molecular imaging with radiotracers

Over the last decade, many promising tumor-specific radiotracers have been developed and evaluated for assessing tumor metabolic changes with PET and SPECT [84, 92, 93] (Table 2).

*Biological bases of metabolic imaging with radiotracers*

- *Energetic metabolism (glycolysis)*

Reprogramming of the energetic metabolism is a fundamental characteristic of cancer. The first discovered metabolic phenotype was aerobic glycolysis (Warburg effect), by which energy generation shifts from oxidative phosphorylation to anaerobic glycolysis, even under normal oxygen concentrations [92]. 2-[^18^F]-fluoro-2-deoxy-d-glucose (FDG) is the radiopharmaceutical most frequently used for clinical PET imaging. FDG is a glucose analog that can be transported into cytoplasm by glucose transporters (Glut). Malignant tumors have a higher metabolic rate and generally express higher numbers of membrane Glut than normal cells. This results in increased uptake of FDG by tumor cells and forms the basis of FDG-PET imaging. In general, higher-grade and less-differentiated tumors are associated with higher uptake of FDG [84, 94–97].

- *Biosynthetic metabolism*

**Fig. 12:**
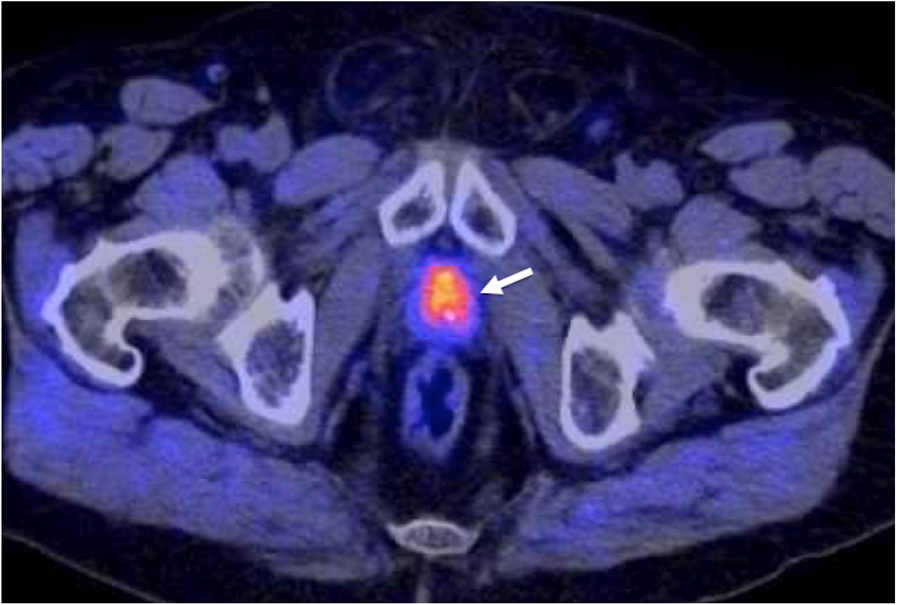
An axial ^18^F-Choline PET/CT image depicted a mass in the prostate bed (white arrow) in a 72-year-old man with a biochemical relapse of a prostate cancer following radical prostatectomy

Malignant transformation is also associated with an abnormal anabolic metabolism due to the increasing growing rate. New radiopharmaceuticals have been developed that are capable of giving more specific tumor information of the changes in tumor biosynthetic metabolism, including increased cellular membrane turnover (Choline [Cho]-PET), altered amino acid and protein synthesis (methionine-PET), increased nucleotide (fluorothymidine [FLT]-PET), and lipid (acetate-PET) synthesis [84, 95] (Fig. 12). Besides, molecular imaging allows the evaluation of tumor-specific metabolic pathways, such as dopamine uptake and metabolism in NETs. All this metabolic information may lead to a better sensitivity and specificity in tumor assessment.

#### Technical features

PET imaging is based on the injection of a radiotracer containing a positron emitter. Positrons annihilate with an electron within milliseconds of its emission, releasing two photons moving in opposite directions, which are detected during PET creating a digital image that represents the distribution of the radiotracer in the body. In the case of SPECT, the patient is injected with a radiopharmaceutical and emits radiation in the form of gamma rays, which are detected by a gamma camera. The combinations of a dedicated PET scanner and multidetector CT (PET/CT) or MRI (PET/MRI) have enabled integrated molecular, functional, and high-resolution morphologic imaging and currently play a critical role in oncology. PET/CT or PET/MRI data result in higher sensitivity and specificity of cancer assessment compared to PET alone. Combined PET/MRI brings the inherent advantages of MRI, including lack of ionizing radiation exposure, increased soft tissue contrast, and the possibility of a multiparametric imaging based on the combination of MR-based imaging techniques.

#### Interpretation guidelines

**Fig. 13:**
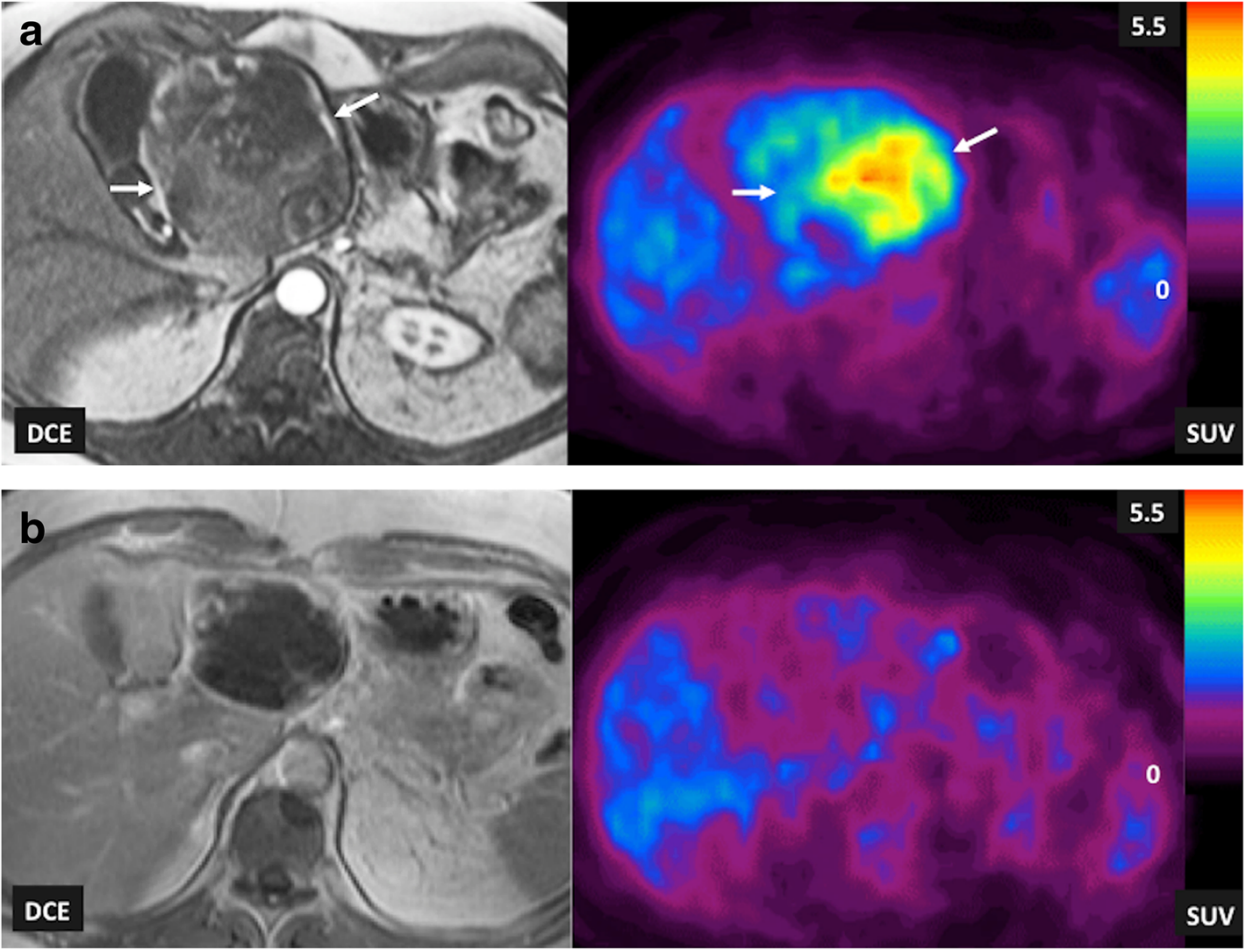
Liver metastasis of a gastrointestinal stromal tumor (GIST) in a 68-year-old man. T1-weighted contrast-enhanced MRI (DCE) images and FDG-PET SUV parametric maps (SUV) acquired pretherapy (**a**) and following the administration of imatinib (**b**) and pretherapy (**a**), imaging findings evidenced a metabolic active tumor with areas of heterogenic enhancement (white arrows). Post-therapy (**b**), the tumor presented almost a total absence of enhancement and a complete metabolic response on PET imaging

Visual interpretation of radiotracer uptake is the basis of the clinical report in PET/SPECT imaging. Absolute quantification would require complex dynamic protocols and repeated measurement of activity concentrations in arterial blood. Thus, normalization approaches have been included in clinical practice. Normalization is based on the measured radiotracer concentration with regard to the injected activity per weight, which results in a semiquantitative index, called standardized uptake value (SUV) (Fig. 13). Qualitative scales have been also used in clinical practice. Lesion uptake is compared with the uptake of a reference tissue uptake (e.g., mediastinum or liver), such in the Deauville five-point scale of lymphomas [96].

#### Clinical value

SPECT and PET have been used for the evaluation of molecular processes in cancer patients. SPECT technology had greater accessibility, lower cost, and availability of a wider range of approved radiotracers. However, PET currently has substituted many existing SPECT oncologic applications due to its superior resolution, speed, and quantitative capability [98, 99].

- FDG-PET imaging

FDG is currently the most used radiotracer in clinical practice. A review of the literature established an average FDG-PET sensitivity of 84% and specificity of 88% across all oncology applications [100]. Besides, reported frequencies of change of patients’ management based on PET findings ranged from 30 to 40% in the literature. PET was associated commonly with the demonstration of greater cancer burden or more anatomic sites involved [101, 102] and the metabolic tumor volume, which combines the dual characteristics of three-dimensional volumetric data and the metabolic activity of tumor based on FDG uptake, has been shown to be a predictor of patient outcome in human solid tumors [103]. Main clinical indications of FDG-PET/CT in oncology include (a) biopsy guidance, (b) evaluating the extent of disease in known malignancies (staging/restaging), (c) therapy planning, (d) predicting pre- and intra-treatment cancer treatment outcomes, (e) establishing tumor prognosis, (f) evaluating tumor response to therapy (Fig. 13), and (g) follow-up to detect cancer recurrence (especially in asymptomatic patients with rising tumor marker levels and those with negative or equivocal conventional imaging findings) [100–102]. Although PET/CT is not frequently used in the setting of tumor diagnosis, differentiating benign from malignant lesions (e.g., in the case of single pulmonary nodules) and searching for an unknown primary are also recognized clinical uses of FDG-PET imaging.

**Fig. 14:**
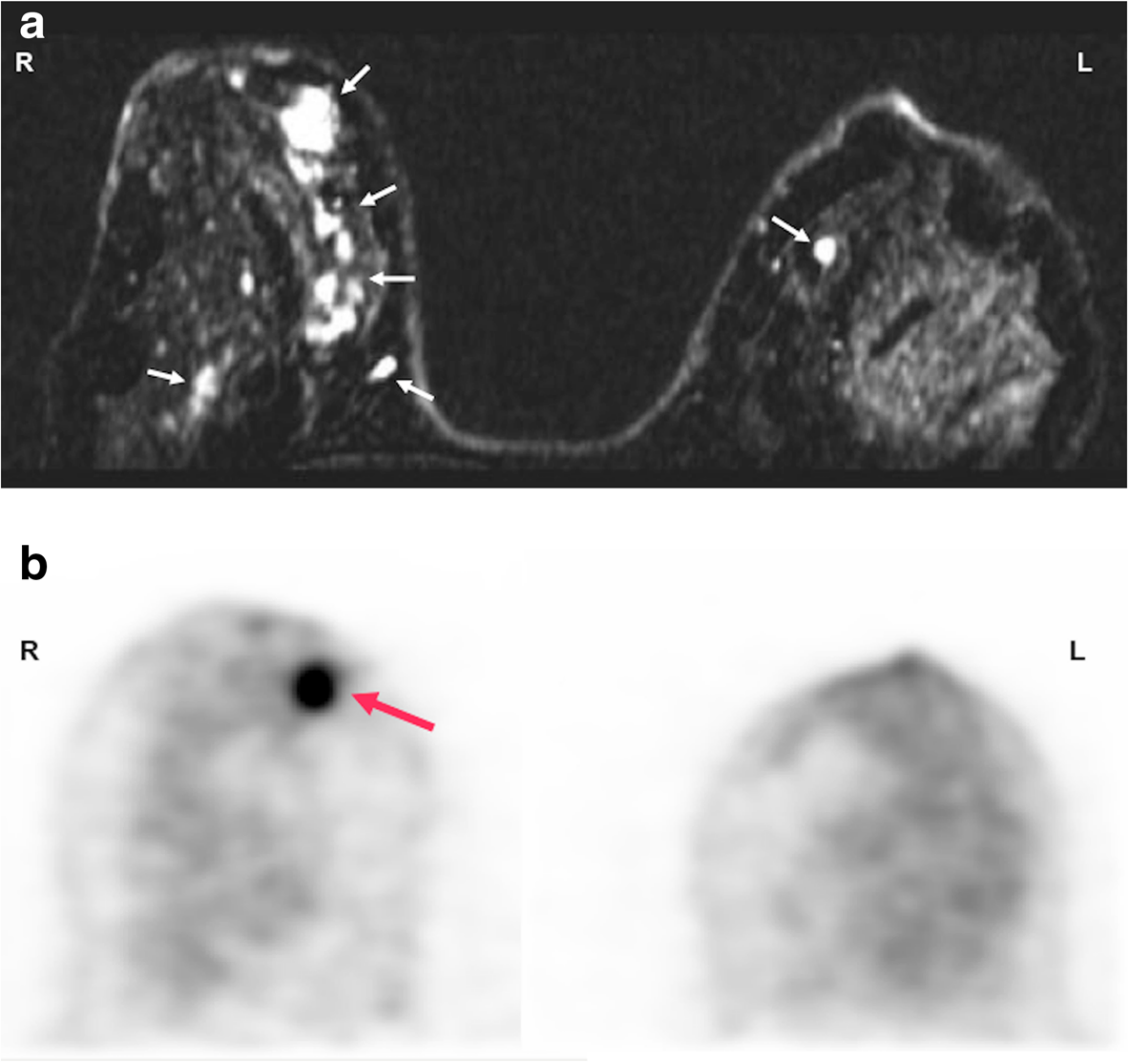
A 48-year-old woman with breast cancer. **a** Axial contrast-enhanced T1-weighted MR image of breasts performed during last week of menstrual cycle showed bilateral multiple nodular areas of enhancement in both mammary glands (white and red arrows). **b** A dedicated FDG-PET exam only depicted a nodular mass (red arrow) with increased FDG uptake in the right breast, corresponding to a invasive ductal carcinoma

New technologies such as dedicated PET imaging devices and PET/MRI technology have been developed to improve cancer imaging. Dedicated PET has been mainly used in breast cancer and brain imaging. Mammography with molecular imaging PET (MAMMI-PET) is a system for dedicated hanging-breast imaging without compression. MAMMI-PET offers higher sensitivity for primary breast cancer lesions comparing to conventional PET/CT [104] (Fig. 14). On its part, PET/MRI technology is being used in those clinical scenarios in which sequential PET and MRI are the standard of care, particularly in brain, liver, bone, or pelvic imaging, but also in children or in those patients undergoing repeated imaging for whom cumulative radiation dose must be kept as low as reasonably achievable and in whole-body staging. However, robust studies demonstrating utility in clinical practice are needed [105–107].

Finally, it is necessary to consider that the value of FDG-imaging in oncology depends on several factors, such as the specific tumor type or the anatomic are of interested. FDG-PET presents recognized limitations, including (a) low sensitivity in well-differentiated/low-grade cancers, tumors with relatively low glucose metabolism (e.g., non-FDG-avid lymphomas, RCC, and bronchoalveolar cell carcinoma), hypocellular cancers (such as mucinous tumors), and tumors with low expression of Glut-1 such as PCa; (b) false positive uptake in benign processes (infection and inflammatory lesions); and (c) increased FDG accumulation in some normal tissues (brown fat) and metabolically active organs (e.g., heart and brain) [91, 99–102].

- Non-FDG PET imaging

**Fig. 15:**
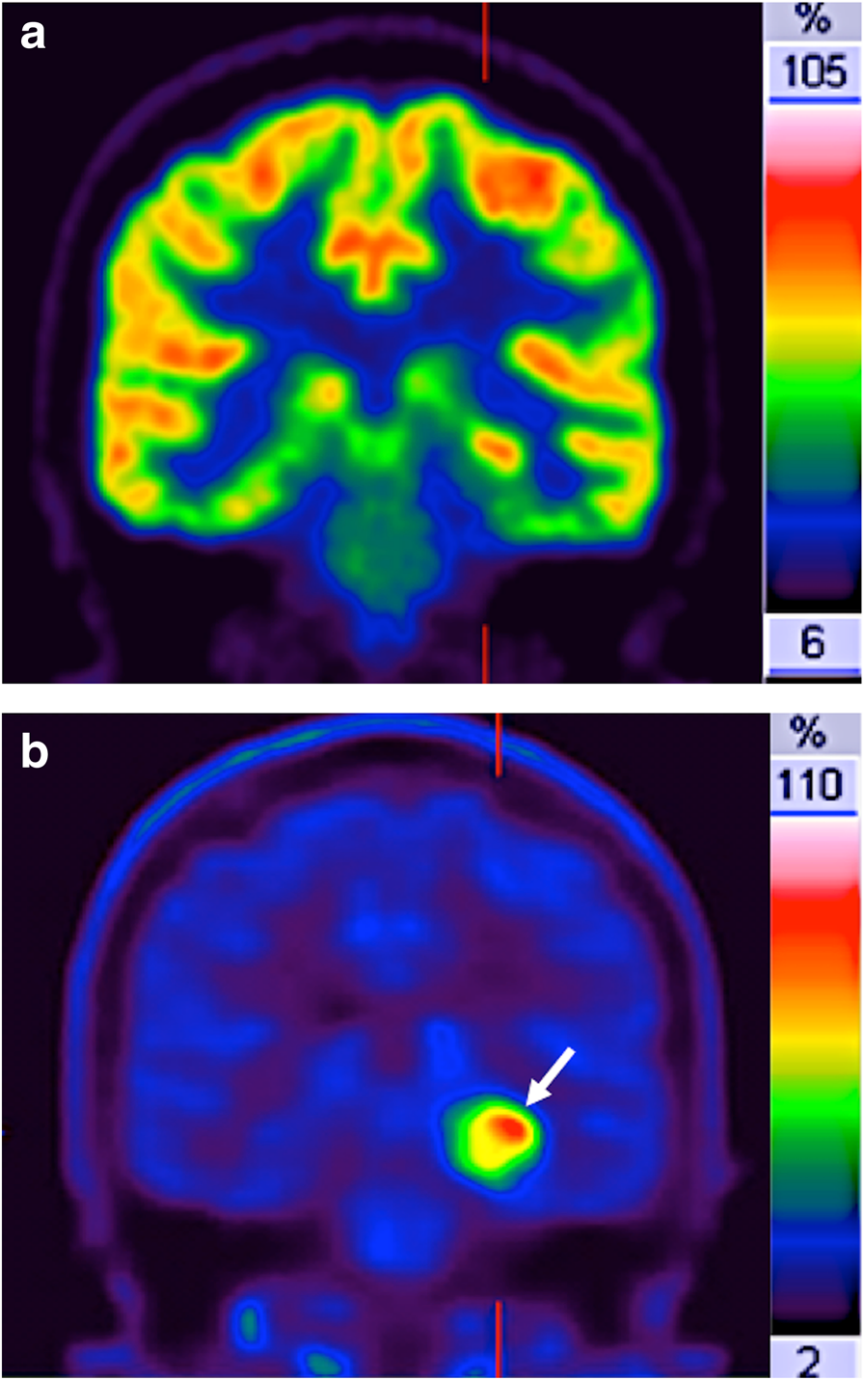
Relapsing brain astrocitoma (white arrow) in a 54 year-old-man. **a** ^18^F-FDG and **b** ^11^C-methionine coronal-reformatted images. Normal uptake of FDG in brain impeded an adequate tumor detection and delineation. However, ^11^C-methionine PET clearly depicted the tumor (white arrow)

Apart of FDG-PET imaging, various other new tracers are gaining a remarkable place in clinical practice for cancer imaging [84, 94]. Malignant transformation is associated with an abnormal Cho (an essential component of phospholipids and cell membranes) metabolism based on both the increasing growing rate and on the upregulation of Cho kinase. The use Cho labeled with ^11^C or ^18^F is mainly focused on the restaging of patients with biochemical failure of PCa. However, the detection rate of Cho-PET varies depending on the site of recurrence, prostate-specific antigen levels, and the presence of hypoxia (which decrease the uptake of Cho-labeled radiotracers). On the other side, ^11^C-methionine may reflect amino acid transport and protein metabolism. This tracer has a main advantage in brain tumor imaging compared with FDG, because there is almost no tracer uptake in normal brain tissue (Fig. 15). ^11^C-acetate is an indirect biomarker of fatty acid synthesis, which is also upregulated in several tumors. In clinical practice, acetate-PET has a major application in imaging tumors in which FDG-PET is of limited use, such as PCa, renal cancer, and hepatocellular carcinoma. Finally, NETs have distinctive biochemical features based on an increased dopamine metabolism, which allow the possibility to image and treat these tumors with specific radioligands [85].

### MR spectroscopy

MRS and MRSI enable in vivo measurements of a complete spectrum of metabolites that are present at millimolar concentration.

#### Molecular and biochemical bases of cancer evaluation with MRS/MRSI

Cancers usually display altered peaks of different biological-meaning metabolites, including Cho, creatine (Cr), lactate (Lac), lipids (Lip), citrate (Ci), etc. The diagnostic importance of a metabolite is depending on the type of tumor and on the organ/anatomical area to be studied [108, 109] (Table 3).

**Table 3:** Main metabolites studied in ^1^H-MRS and their biological significances

Metabolite	Biological meaning	ppm	Decreased values	Increased values
Cho (total Cho-containing compounds)	A metabolic marker cell membrane synthesis and repair. Related to cell density and membrane integrity	3.22		Due to cell proliferation and to breakdown of cell membranes. Higher Cho levels are shown in higher grade tumors compared with lower-grade tumors
Cr (from both creatine and phosphocreatine, often called referred to as total creatine peak)	Reflects “cellular energetics”	3.02	Decreased phospocreatine (PCr) is an inconstant finding in tumors. Represents a low- energy status of glycolisis in primary tumors (high grade gliomas) and in metastatic tumors is the effect of the lack of PCr in most tissues.	
Lac	Glycolysis Usually lactate is present only in minimun amounts (i.e., in the brain) and is not depicted using the normal spectroscopic techniques.	Doublet peak at 1.33 ppm		Increased Lac is the effect of the high rate of glycolisis. Lac is an end product of glycolysis and increases rapidly during hypoxia It accumulates in necrotic or cystic areas
Lip	May indicated tumor necrosis or voxel contamination by diploic space fat, scalp and subcutaneous tissue	0.9 and 1.3ppm		The rise of Lip is detected in various cellular processes such as necrosis, growth arrest, inflammation, malignancy and apoptosis.
Specific metabolites in brain H-MRS				
NAA	A marker of neuronal and axonal density and viability The exact role of NAA in human brain metabolism is uncertain. It is postulated to be involved in lipogenesis pathways Largest peak in a “normal” H- MRS brain spectrum.	2.02	Absence of neurons and axons in most tumors but also in white matter diseases (i.e., multiple sclerosis)	
mI	A glial marker located in astrocytes Involved in osmoregulation and volume regulation	3.56		It is a relative marker for low-grade gliomas. Reduced in high-grade tumors
Specific metabolites in Prostate H-MRS				
Ci	Normal human prostate gland accumulates and secretes extraordinarily high Ci levels	2.6		In prostate cancer Ci levels fall due to consumption of Ci to supply energy to proliferating cells)

*Ala* alanine, *Cho* choline, *Ci* citrate, *Cr* creatine, *Lac* lactate, *Lip* lipids, *mI* myo-inositol, *NAA* N-acetylaspartate, *ppm* parts per million

#### Technical features

Spectroscopy uses the free induction decay (FID) following a radiofrequency to obtain metabolite information. One-dimensional Fourier transforms of the FID gives spectra with peaks, whose areas are proportional to the abundance of metabolites. MRS provides information about the chemical environment of the nuclear spin, which depends on the neighboring nuclei and overall chemical structure. This chemical environment changes the B0 magnetic field to which this nucleus would normally be exposed in the magnet, and these resonance frequency differences (chemical shift) allow that metabolites can be distinguished from each other. Every molecule exhibits its own “fingerprint” on the chemical shift spectrum. The exact chemical shift of a metabolite (expressed in parts per million, ppm) is independent of the applied field strength and characteristic of that nucleus, aiding accurate peak identification. Clinical MRS mainly uses endogenous signals from ^1^H or ^31^P. ^1^H-MRS is the most commonly used technique because it produces the strongest signals (due to its abundance). On its part, ^31^P MRS can measure the relative intracellular concentrations of several phosphorus metabolites. However, it is technically more complex and requires special hardware. Spectroscopic measurements generally give a static picture of tumor metabolism. In contrast, hyperpolarized MRSI offers a dynamic evaluation of tissue metabolism. Hyperpolarized MRSI increases the signal-to-noise ratio (SNR) achieved with MR. When exposed to the magnetic field, the magnetic dipole vectors of MR-active nuclei (such as ^13^C) are aligned either parallel or antiparallel with the external field, and there is usually only a small excess of parallel spins (the polarization percentage). However, higher polarization values can be reached for short periods of time via hyperpolarization, producing larger vectors and facilitating high-SNR data and shorter acquisition times in MRS imaging acquisitions [110].

#### Interpretation guidelines

In clinical practice, tumor spectroscopic phenotype can be evaluated in three ways: (a) qualitative evaluation is performed by merely checking absence, presence, or change of certain metabolites; (b) semiquantitative evaluation is based on two different approaches: the calculation of metabolite ratios (e.g., Cho + Cr/Ci ratio, which correlates significantly with the probability of malignancy in prostate) or the evaluation of the area under the metabolite peaks of interest; and (c) finally, absolute quantification of the concentration of a metabolite with the use of a reference standard for calibration.

#### Clinical value

Spectroscopy of cancer can characterize critical tumor metabolic pathways (including membrane turnover, lipids, and energy metabolism) and can depict specific tissue markers, such as *N*-acetyl-aspartate (NAA) (a specific neuronal marker) or Ci. MRS technique in tumors is mainly based on detecting the elevation of certain metabolites (such as Cho) or the absence or decrease of normal metabolites (e.g., Ci in the prostate) [108, 109–112]. However, its technical complexity, long measurement times required, and frequent low-quality spectra have limited its widespread use.

- *Hydrogen MRS*

Characterization of intracranial tumors is a challenge for imaging. In this setting, main ^1^H-MRS studies have focused on the assessment of intracranial tumors [113]. Spectroscopy offers additional information related to tumor proliferation and metabolic changes or neuronal damage, which can be used to establish noninvasively the diagnosis and grading of brain tumors. An increased Cho peak along with decreased NAA is an important diagnostic feature in brain tumors. Besides, increased Lip seems to be secondary to necrosis and membrane breakdown. Cancer cells also rapidly metabolize glucose to form Lac and its levels appear to correlate with grade and type of tumor in the brain. Finally, a myo-inositol (MI) peak is typically present in glial tumors at 3.5 ppm chemical shift. ^1^H-MRS has shown clear advantages for the assessment of central nervous system neoplasms including differentiating between tumors from other lesions, characterizing types and grades of tumors, offering prognostic data, planning therapy with delineation of tumor involvement and definition of the target volume for radiation therapy, monitoring tumor response, and detecting tumor relapse.

Technical advances allowed the use of MRS in other organs, mainly prostate and breast [108]. PCa is characterized by combinations of elevated Cho and reduced Ci. ^1^H-MRSI in prostate can improve tumor detection, localization, and staging; assessment of tumor aggressiveness; and evaluation of tumor response. A meta-analysis of the literature to assess the accuracy of MRS in diagnosing PCa evidenced that the pooled weighted sensitivity and specificity varied depending on the (Cho + Cr)/Ci ratios used, ranged between 64–82 and 68–86%, respectively. Moreover, a positive correlation was found between these ratios and the pathologic Gleason score with a large proportion of non-significant tumors (Gleason score ≤ 6) that do not present abnormal metabolite ratios [114]. However, at present, the clinical use of MRS in prostate has been reduced due to its technical complexity. Thus, version 2 of the Prostate Imaging-Reporting and Data System (PI-RADS v2) does not include the use of MRS.

**Fig. 16:**
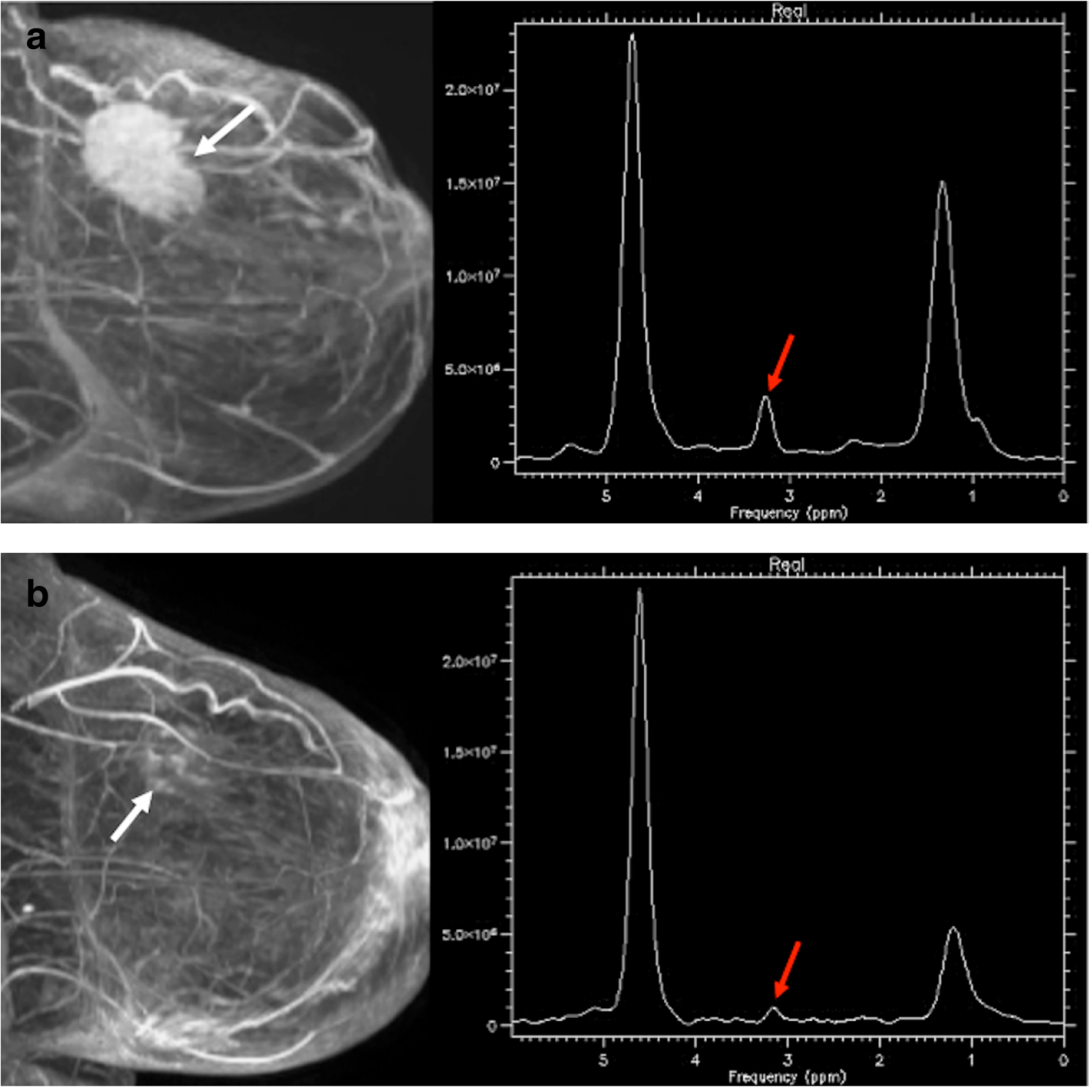
A 56-year-old woman with an invasive ductal carcinoma of the breast. Pretherapy (**a**) the sagittal reformatted maximum intensity projection (MIP) image from DCE-MRI (left) demonstrating a 5-cm enhancing mass (white arrow). MR spectrum (right) depicts a marked choline peak (red arrow). Images obtained after chemotherapy and anti-HER2 therapy with trastuzumab (**b**) evidenced a residual lesion. Sagittal reformatted maximum intensity projection (MIP) image from DCE-MRI demonstrated discrete dots of enhancement (white arrow), while MR spectrum showed a decreased choline peak (red arrow). Tumorectomy revealed residual fibrosis without tumor cells

In breast tumors, an increased Cho peak has been described in malignant breast lesions (Fig. 16). A meta-analysis of the diagnostic performance of single-voxel ^1^H-MRS of the breast reported a pooled sensitivity ranged between 71 and 74%, and a pooled specificity between 78 and 88%. However, in the case of non-mass lesions or small masses (between 5 and 10 mm in diameter) or foci (< 5 mm in diameter), spectroscopy was scarcely useful [115].

- *Phosphorus MRS*

Malignant transformation has been found to alter the profile of the Cho compounds (related to membrane turnover) and the signals from energy metabolites such as nucleoside phosphates and phosphocreatine. Although ^31^P MRS may monitor these metabolic changes, its clinical use has been limited due to technical complexity and low sensitivity of MR systems [111, 112].

- *Hyperpolarized MRSI*

**Fig. 17:**
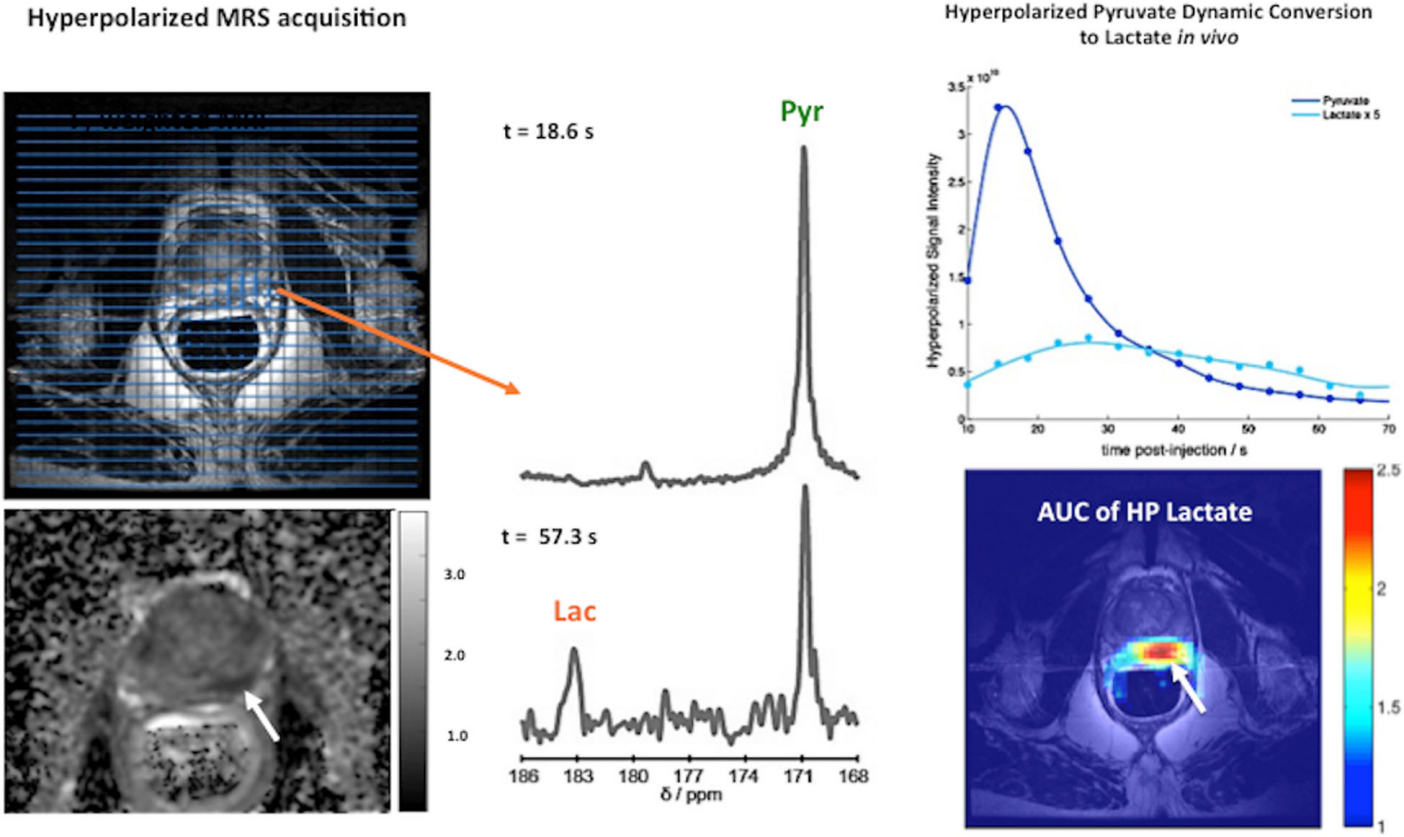
A 71-year-old man with a Gleason 7 prostate cancer (PSA 6.05). A suspicious area with low ADC values was depicted in the left peripheral area (white arrow). The hyperpolarized ^13^C spectra and the time course for the dynamic conversion hyperpolarized [1-13C] pyruvate to lactate following the injection of hyperpolarized pyruvate were revaluated (top, right). Note the change of the MR spectra with the pyruvate quickly reached a maximum at *t*: 57.3 s (bottom, middle) before being converted to lactate. Compared with the spectrum at *t*: 18.6 s (top, middle). [Images courtesy of Kayvan R Keshari, PhD. Laboratory Head. Memorial Sloan Kettering Cancer Center]

Currently, there is a limited clinical use of ^13^C hyperpolarization in clinical studies and the majority is focusing on the pyruvate metabolism in PCa (Fig. 17). Hyperpolarized [1–^13^C]-pyruvate has demonstrated increased lactate labeling in tumors and decreasing metabolism to bicarbonate [116].

### Imaging tumor proliferation

Sustained proliferation is a fundamental part of cancer development and progression. The imaging of tumor proliferation may provide valuable information for tumor diagnosis and characterization and early assessment of the response to therapy.

#### Biological bases of tumor proliferation

EGFR is commonly upregulated in cancers. EGFR over-activates downstream pro-oncogenic signaling pathways, including the Ras-Raf–mitogen-activated protein kinase (MAPK) pathway and the PI3 kinase (PI3K)–Akt–mechanistic target of rapamycin (mTOR) pathway. These pathways activate cell growth, proliferation, and survival.

#### Imaging approaches to tumor proliferation

Noninvasive imaging-based assessment of cellular proliferation in cancer is mainly based on radiotracers that track the thymidine salvage pathway of DNA synthesis.

- *PET imaging of tumor proliferation*

[^18^F]-3-fluoro-3-deoxythymidine (FLT) is a nucleoside-analog imaging agent that presents intracellular accumulation of the tracer during the S-phase of the cell cycle through the action of the thymidine kinase-1 (TK1). Since this kinase is primarily expressed in dividing cells, FLT uptake is essentially limited to proliferating cells. FLT uptake has been shown to correlate with Ki-67 expression, a classic marker of tumor proliferation [117]. FLT has been rarely used for tumor response evaluation due to the limited knowledge of the factors determining FLT uptake and therapy-induced changes of its retention. Nevertheless, published data suggest that decreased radiotracer uptake generally reflects the effects of anticancer therapies, although it should be taken into account that drugs impacting TK1 (such as antifolates) may induce a flare effect.

Other radiotracers (Cho-PET) and different imaging techniques (MRSI or DWI) may also offer an indirect assessment of tumor proliferation. Cho metabolism is involved in the synthesis of Lip required for cell membrane turnover in tumor proliferation. In this setting, MRSI Cho peak and Cho-PET uptake may indirectly evaluate the cell proliferation. Thus, a number of studies have found significant correlations between the SUV of Cho-PET or Cho peaks on MRSI and Ki-67 proliferation scores. Changes in Cho metabolism are being used in oncology for diagnosis, prognosis, and monitoring response. However, it is also necessary to consider that Cho is not a specific cancer tracer (e.g., normal testicles show elevated Cho peaks and granulomatous LNs may present increased uptake on Cho-PET).

DWI may also reflect other histopathological features, such as proliferation potential. However, based on a recent meta-analysis, the correlation of DWI-derived parameters and tumor proliferation based on the expression of cell proliferation markers (e.g., Ki67) showed a great variability depending on tumor type [118, 119].

### Evaluation of tumor vasculature-angiogenesis

Tumor angiogenesis is the process of developing new blood vessels in order to supply oxygen and nutrients to support the growth of tumors. Angiogenesis is a key cancer hallmark required for invasive tumor growth and metastasis development and constitutes a basic target in the therapy of cancer. Imaging modalities used to evaluate tumor neovasculature mainly include CT, MRI, or US [120–125]. All of them have strengths and weaknesses regarding availability, sensitivity, accuracy, biological significance of the obtained data, technical reproducibility, methods for image quantification, provided parameters, and the anatomical areas that can be imaged.

#### Biological bases of angiogenesis

Most solid tumors arise as avascular cellular masses that exclusively depend upon oxygen delivery by diffusion. When tumor size exceeds oxygen diffusion distances, regions of hypoxia will develop within the mass. This feature causes the secretion of angiogenic factors (particularly the vascular endothelial growth factor, VEGF), which activate the angiogenic switch. However, tumor vasculature is highly abnormal. Tumor vessels are often larger but less numerous and more ineffective than those of normal tissues. Besides, tumor blood vessels are heterogeneous and abnormal with regard to organization, function, and structure. The combination of these vascular characteristics causes that the overall BF tended to be significantly lower in tumors and the subsequent development of hypoxia. Unfortunately, imaging techniques used in clinical practice usually check the effects of angiogenesis on tumor vasculature, not the angiogenic process itself [120–126].

#### Technical features

**Fig. 18:**
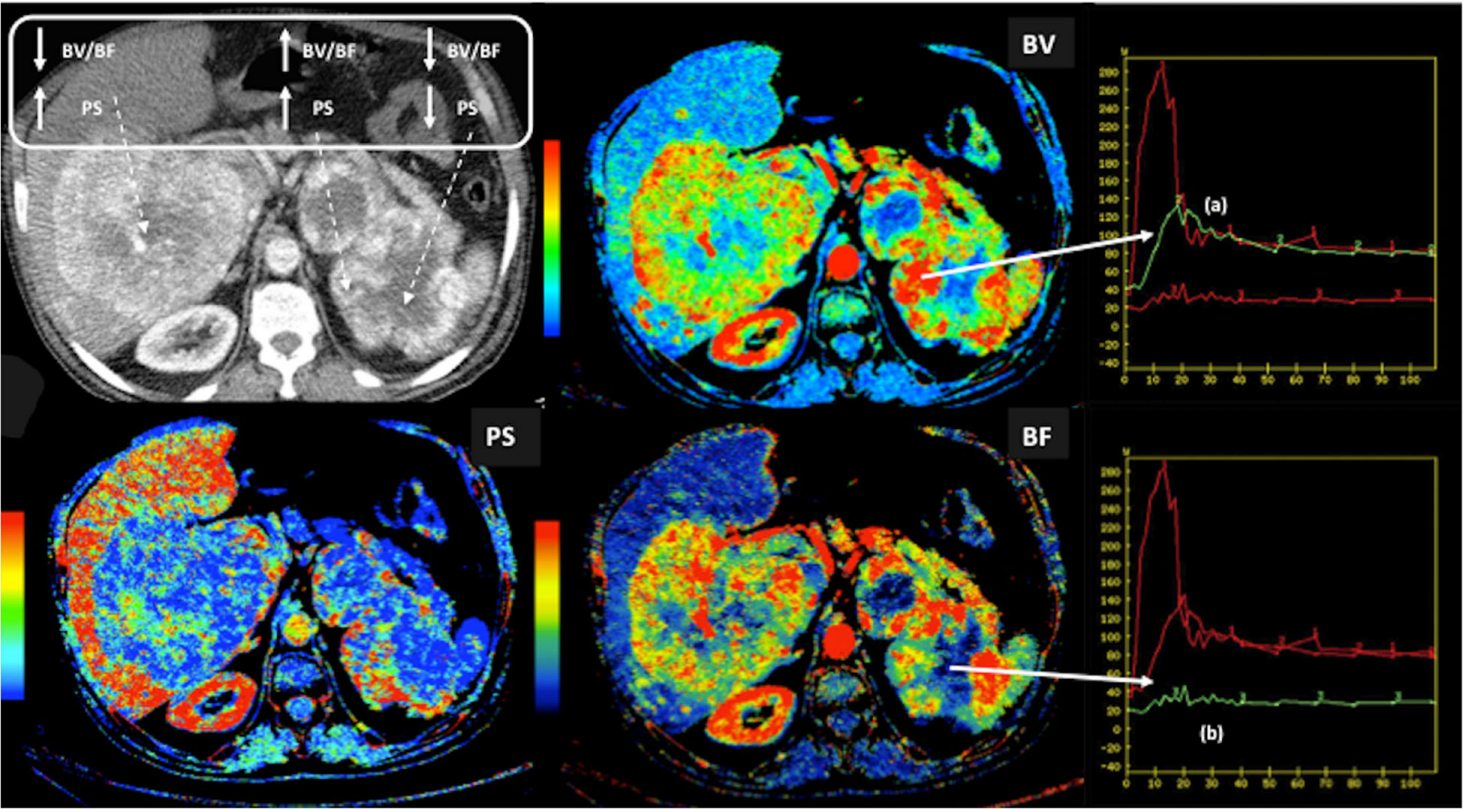
Perfusion CT exam in a 42-year-old man with a metastatic renal carcinoma. Blood flow (BF), blood volume (BV), and permeability (PS) parametric maps evidenced the heterogeneity of the functional status of the vasculature within the tumor with several combinations of the obtained parameters (top-left) and clear differences between the time density curves coresponding to the peripheral areas of viable tumor with increased BV, BF, and PS (**a**) with a wash-in/wash-out pattern and the central necrotic necrotic areas (**a**)

**Fig. 19:**
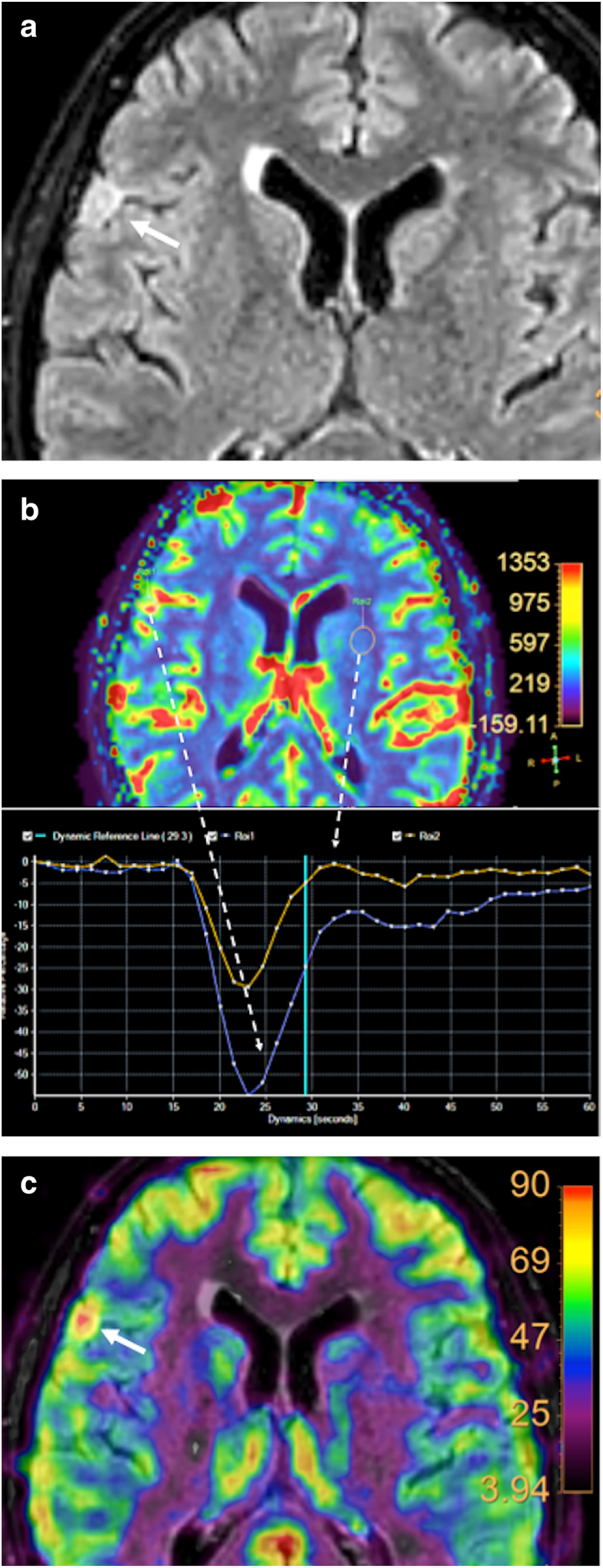
Brain perfusion in a 55-year-old woman with a meningioma. **a** An axial fluid attenuation inversion recovery (FLAIR) image of the brain demonstrated a hyperintense extra-axial insular lesion (white arrow). Both (**b**) dynamic susceptibility contrast (DSC)-MRI and (**c**) arterial spin labelling (ASL) parametric maps demonstrated and increased perfusion in the mass. Comparison to normal brain parenchyma evdenced a higher decrease in MR signal intensity curves during the first-pass of the contrast bolus (**b**, blue curve)

Imaging plays a central role in the clinical evaluation of angiogenesis. Anatomical imaging remains the mainstay for tumor evaluation pinpointing sites of enhancement and evaluating the degree of enhancement. New technologies, such as dual-energy CT, may have an added value over conventional CT imaging for tumor assessment. In this setting, quantitative iodine concentration maps obtained with spectral CT have shown good correlations with conventional CT perfusion measurements in different tumor types [127]. Imaging techniques can also characterize functional abnormalities of vessels and can assess vascular heterogeneity. Different imaging techniques with and without the use of exogenous contrast media have been used for the functional evaluation of tumor vasculature. DCE techniques including CTP, DCE-US, DCE-MRI, or DSC-MRI are the most common imaging methods for assessing tumor vasculature [120–126]. These imaging techniques acquire a series of images through a region of interest before, during, and after the intravenous injection of a contrast agent. DCE techniques may provide information related to tumor vessels function (perfusion or permeability). Data obtained depend on multiple features including imaging technique, the type of contrast agent, and the method of analysis. On its part, IVIM and ASL constitute the main non-contrast-enhanced imaging techniques for the evaluation of tumor vasculature. CTP evaluates temporal changes in tissue attenuation following the intravenous administration of contrast media. In this context, iodine concentration changes may represent an indirect reflection of tissue vascularity and vascular physiology [126] (Fig. 18). On its part, DCE-US with gas-filled microbubbles offers potential for the evaluation of tumor perfusion. Microbubbles flood across tumor capillary bed and the gas they contain contracts and expands under the alternating higher and lower pressure phases of the US beam, producing harmonics that can be detected for extracting kinetic characteristics from DCE-US echo intensity (which is directly related to local vascularization and contrast-agent concentration) versus time data [128]. DCE-MRI calculates tissue perfusion parameters by evaluating T1 shortening induced by a gadolinium-based contrast agent bolus as it distributes in the tumor vasculature and leaks across permeable vessels. The resulting signal intensity–time curve reflects a composite of tissue perfusion, vessel permeability, and extravascular-extracellular space and can be analyzed to derive a number of potential biomarkers of the vascular microenvironment. DSC-MRI or T2*-weighted DCE is based on the local magnetic inhomogeneities that arise on the boundaries between structures that differ in their magnetic susceptibility, leading to signal reduction on T2 or T2*-weighted gradient-echo sequences. The transient signal loss induced by the pass of the contrast agent across tissue vascular network is proportional to the local BV and BF. DSC studies are basically limited to the evaluation of brain tumors. Finally, ASL is based on the principle of magnetically labeling inflowing arterial blood protons inverting the bulk magnetization of the blood water protons prior to their entry into the tissue of interest. A control image, in which the blood water magnetization is not inverted, is also acquired. The signal difference between labeled and control images will be proportional to BF. Quantification allows for regional and global assessments of perfusion. ASL is being applied in brain imaging (Fig. 19) [129].

#### Interpretation guidelines

Many features need be considered in the acquisition and analysis of DCE exams, including the organ studied and the clinical setting. The characteristics of perfusion data acquisition (temporal resolution, length, etc.) and the model of analysis of enhancement kinetics directly influence the calculation of vascular parameters. Analysis and interpretation of DCE-imaging data can be performed with different approaches [120, 121]:

- Qualitative analysis: Visual assessment of pre- and postcontrast images, or of the shape of the time-intensity curves (TIC) represents the most simple way of analysis. Curve characteristics, including the speed of the filling phase, the maximum peak intensity, and the morphology following the peak of enhancement (persistent increase, plateau, or washout), are evaluated. This approach has shown clinical utility for the characterization of breast, soft-tissue, and prostate tumors, although does not produce a quantifiable index.
- Semiquantitative analysis: This type of evaluation is based on performing a direct analysis of changes in SI using semiquantitative indices. Main descriptors that characterize the shape and structure of the curves include time to peak enhancement, initial area under the curve (IAUC), maximum enhancement, etc. These parameters have been used in the clinical evaluation of brain, breast, and prostate tumors. Unfortunately, they do not present a clear defined relationship with tumor physiology.
- Quantitative analysis: The combination of DCE imaging with mathematical modeling of the contrast agent kinetics enables quantitative assessment of the structural and functional changes in tumor microvasculature. Measured TICs must be converted into concentration of contrast–time curves and modeled in a pixel-wise fashion to create functional maps of vascular parameters. In general, the complexity of the quantitative analysis and the lack of consensus have limited its applications to clinical trials in academic centers.

#### Clinical value

The angiogenic phenotype shows a great heterogeneity among different type of tumors and even in the same tumor type or in different regions of the same tumor. Imaging-based phenotypes are important features for the decision-making process in oncology, including tumor diagnosis, characterization and grading, biopsy guiding, staging, prognostic biomarker and response prediction, therapy planning, assessment of treatment response, detection of relapsing tumor, and development of new cancer drugs. Tumor vasculature provides an attractive target for anticancer therapies [120, 121, 126]. DCE-imaging is now frequently used in early clinical trial assessment of angiogenic inhibitors [43, 126, 130]. In these patients, therapy response is associated to a significant decrease in perfusion and permeability-related parameters. Moreover, an early reduction in vascular parameters following therapy is usually associated to an improved patient’s outcome.

### Imaging cell death in cancer

Imaging programmed cell death would be useful both in clinical care and in drug development.

#### Biological bases of cell death

Cell death has been classified, on the basis of morphologic criteria, as programmed cell death (apoptosis) or non-regulated (necrosis) [131]. Necrosis is characterized by loss of plasma membrane integrity, which frequently causes marked inflammation, tissue destruction, and fibrosis. On its part, apoptosis is accompanied by a series of complex morphological changes, including cell shrinkage, chromatin condensation, and formation of apoptotic bodies. Apoptotic bodies are subsequently ingested by adjacent cells and phagocytes without provocation of an inflammatory response. Apoptosis is also characterized by several molecular alterations, including the externalization of phosphatidylserine to the outer leaflet of the plasma membrane bilayer and the activation of caspases [132]. Phosphatidylserine exposure by dying cells has been evaluated with radiolabeled annexin V. However, it has not become routine in clinical practice.

#### Technical features

Radiotracers and modalities for the imaging of apoptosis include phosphatidylserine-binding agents (annexin V) and MR-based imaging with the assessment of mobile lipids and Cho with ^1^H-MRS and DWI [131–134] (Figs. 6 and 7).

#### Clinical value

There is a limited use of clinical imaging in the evaluation of cell death, although MRI seems to be a promising tool. Apoptosis and necrosis exhibited different changes on MR exams including T1- and T2-weighted sequences, DWI, and MRS [133, 134]. Necrosis secondary to therapy is accompanied by an early increase in ADC values, while apoptotic treatments do not cause significant ADC changes [133]. In the case of ^1^H-MRS, this technique demonstrates apoptosis- and necrosis-specific changes on cell mobile Lip peaks [132].

### Imaging of cancer heterogeneity

Tumors are biologically heterogeneous at the morphologic, histological, and genetic levels. Imaging has begun to map and track the presence of phenotypic heterogeneity between (intertumor) and within tumors (intratumor) of a given patient.

#### Biological bases

**Fig. 20:**
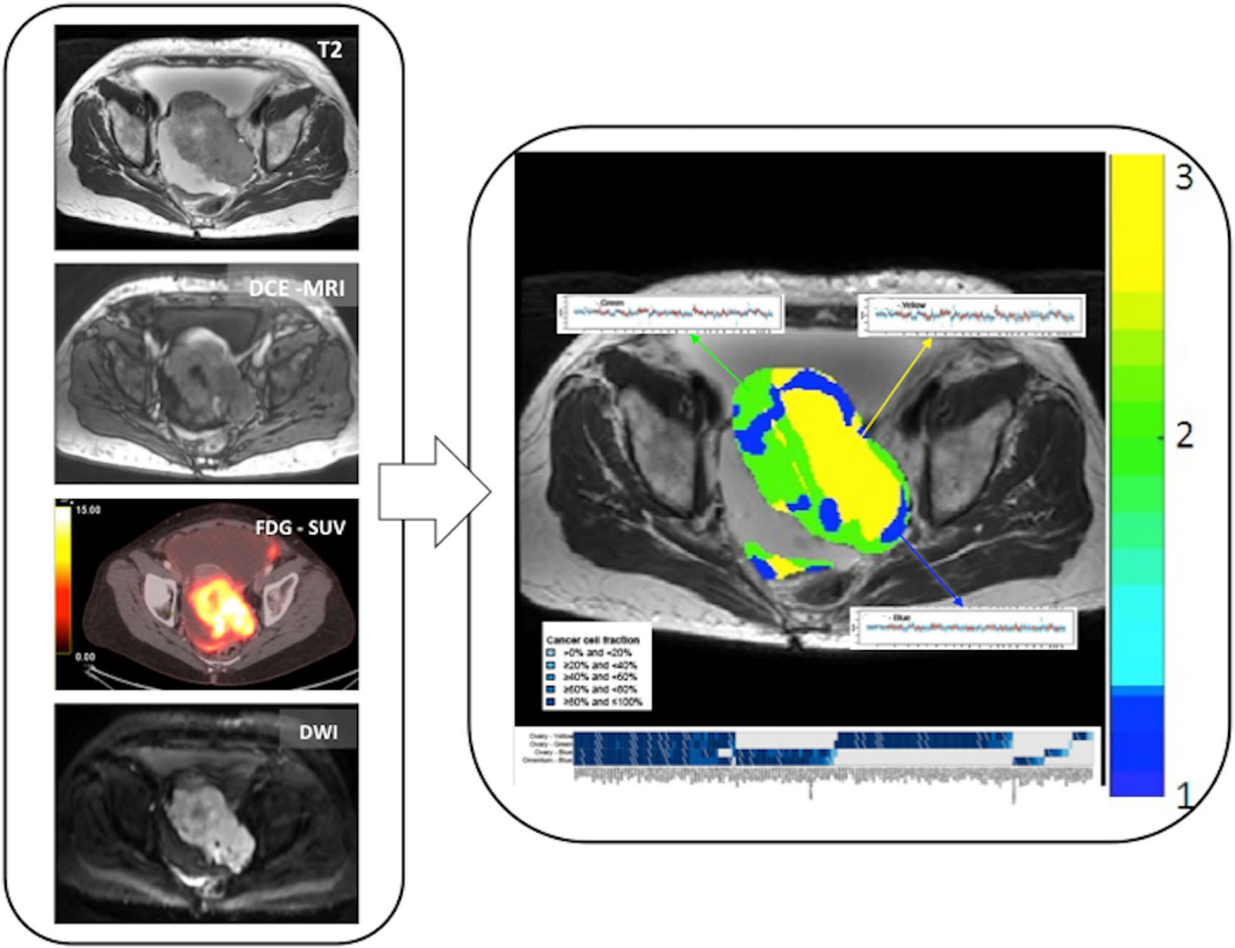
A 70-year-old woman with high-grade serous ovarian cancer who underwent FDG-PET/CT (standardized uptake value map of the fluorodeoxyglucose uptake [FDG SUV] is showed) and MRI, which included T2, diffusion (DWI), and dynamic contrast-enhanced (DCE) sequences (images on the left) showing a primary ovarian mass as well as multiple peritoneal/serosal implants including in the cul-de-sac. A composite “habitat” map of the implant in the cul-de-sac was performed (right) containing three clusters derived from PET and MRI data. Tissue sampling from each cluster represented by a different color on the habitat map demonstrated genomic heterogeneity underpinning the phenotypic heterogeneity observed on imaging

Genetic variation is undoubtedly the most established foundation of both intra- and intertumor heterogeneity. However, other features such as epigenetics or tumor microenvironment also play a role on heterogeneity [135]. Intertumoral heterogeneity has resulted in the classification of tumor subtypes, which are characterized by distinct morphology, functional, molecular and genetic profiles, and expression of specific markers. On its part, intratumoral heterogeneity manifests as variations within the tumors (Fig. 20). Distinct tumor genotypes and/or phenotypes usually associate divergent biological behaviors, and this circumstance may have prognostic significance and may influence response to therapy. In general, the degree of intratumor heterogeneity tends to increase as tumors grow and greater heterogeneity tends to be associated to a relatively poor clinical outcome and resistance to therapy.

#### Technical features

Tumor spatial and temporal heterogeneity is a critical oncologic feature that can be reflected in medical images and interpreted based on different approaches: (1) evaluating simple qualitative descriptors (e.g., tumor margin or shape); (2) quantifying parameter distributions with histogram-based analysis (discarding spatial relationship between voxels and treating data as a list of continuous variables); (3) quantifying spatial complexity (e.g., texture, fractal, or cluster analysis); (4) grouping voxels with common biological features (parcellation); or (5) analysis assessing quantitatively the spatial distribution of parameters (e.g., parametric maps) (which tend to derive average parameter values), etc. [3, 135–139].

#### Clinical value

The advantage of diagnostic imaging techniques in the assessment of tumor heterogeneity is their noninvasive nature and the fact that the whole tumor may be evaluated, whereas histological techniques are invasive and limited to a discrete set of tumor samples. There is a growing evidence that qualitative or quantitative evaluation of heterogeneity presents clinical advantages for screening, diagnosis, grading, staging, and assessment of response therapy in tumors [138]. Unfortunately, many parameters, such as kurtosis, have no clear biological correlate making biological validation difficult. Moreover, in order to improve a global assessment of tumor heterogeneity, whole-body imaging techniques (e.g., PET or WB-DWI) offer an ideal solution as they allow a global assessment of tumor burden.

## Future trends of imaging in cancer

**Fig. 21:**
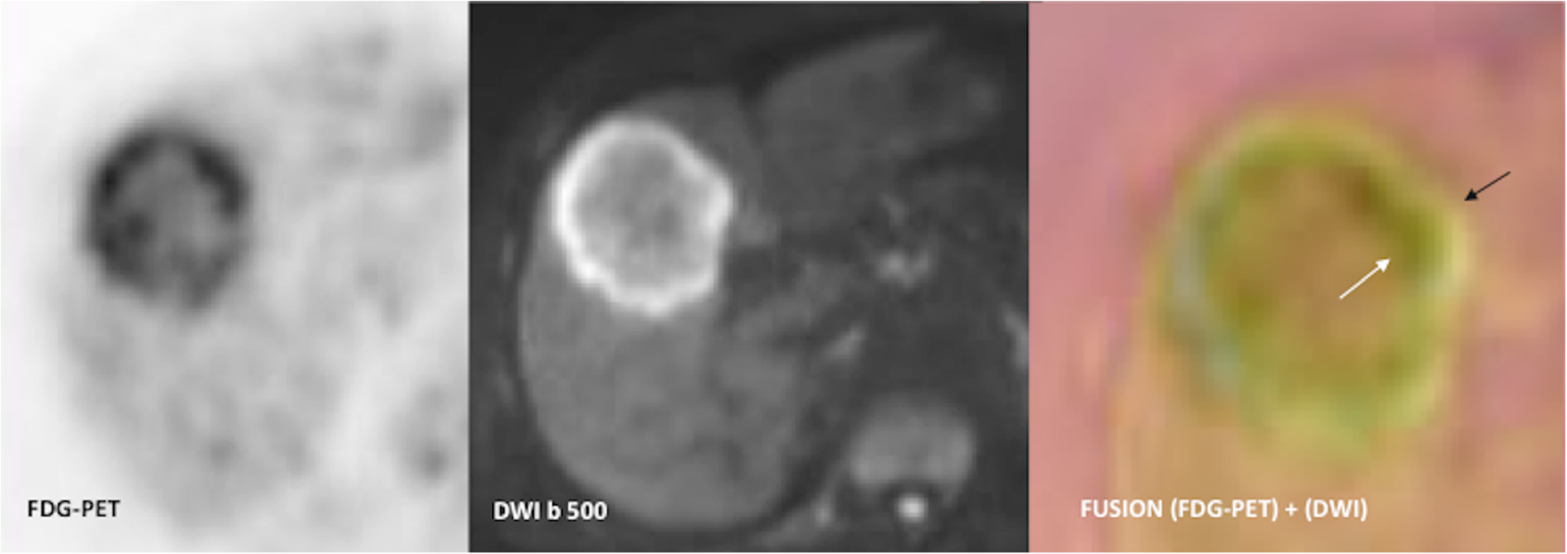
Colorectal cancer liver metastasis in a 56-year-old man. FDG-PET (left) and b500 diffusion-weighted (middle) images demonstrated the presence of a mismatch between the obtained parameters. Note that the size of the FDG abnormality is smaller than the diffusion one (black arrow) (right). Tumor biology may explain this feature. Higher FDG uptake occurs at the edge of the necrotic cavity (white arrow) which is of relative low SI on b500 image. The edge of the necrotic cavity usually represents an area of relative tumor hypoxia, which may promote a high metabolic activity (vascular metabolic-mismatch). On its part, the periphery of the mass generally presents good perfusion and it is the most cellular area of the tumor, explaining the restricted diffusion at this level

There are extraordinary future opportunities for imaging techniques in tumor biology evaluation, including the development of imaging biomarkers and radiomics/radiogenomics, the use of multiparametric analysis and artificial intelligence, and theranostic. To date, most research has been focusing on validating biomarkers extracted from tissue or blood samples, which has improved patient stratification and assisted oncologic drug development. Imaging techniques can evolve into clinically useful biomarkers for tumor assessment and evaluation of therapy response. The advantages of imaging are its versatility, its widespread disponibility, its capability of evaluating whole tumor burden, and its relatively noninvasive nature [140–144]. Adequate quantification of imaging biomarkers is of paramount importance when extracted data are going to be used in patient management. In this setting, data must be reproducible and the technology used to extract them must be standardized. Biomarkers precise a complex process of validation and qualification [140, 143]. On the other side, in recent years, imaging has been boosted by the technological development generating a large volume of data. Such information has increased in complexity and may offer prognostic value and may reveal meaningful information for decision-support in cancer diagnosis and treatment. Radiomics refers to the extraction and quantitative analysis of tumor characteristics from medical images. On its part, radiogenomics investigates the relationship between imaging features and gene expression. The -omic approach is based on numerical calculus and computer science methods, allowing the management and analysis of a very large number of variables for each sample and modality. There is a rapid increase in the number of publications that have highlighted the utility of imaging -omics in many different tumor types and based on different imaging techniques [145–155]. Radiomics and radiogenomics approaches may show clinical utility for assisting in cancer diagnosis, assessment of tumor aggressiveness, response assessment, and evaluation of patients’ outcome. Integrating (quantitative) imaging data with other relevant information (clinical, pathological, etc.) and multi-omics (genomics, proteomics, and metabolomics) will be essential for unraveling tumor heterogeneity and making real-time clinical decisions for patients in personalized medicine. However, this process still necessitates improvement and standardization in order to achieve routine clinical adoption. In this scenario, computers may be useful tools for the assessment of tumor characteristics and for the evaluation of therapy response. Computers can learn (machine learning) to extract meaningful patterns (including patterns that are beyond human perception) by processing massive datasets (big data) through mathematical models (algorithms). Machines can also correct algorithm mistakes by training. Machine learning algorithms are just useful components of computer-aided diagnosis and decision support system in oncology. Imaging representation and interpretation of tumor biology will require computational models to understand and predict the complex nonlinear dynamics that result in combinations of imaging features [156–158]. 3D printing is also an emerging computer-based technique that may be useful in oncology for research, surgical planning (using an exact 3D model of the patient’s organs to practice a procedure), device designing and manufacturing, and tissue or organ replacement [159]. The analysis of multi-dimensional imaging datasets is also increasingly required for imaging tumor phenotype. The correlation between imaging features obtained with different techniques must be explored for understanding the underlying tumor biology (Fig. 21). Significant differences in vascular, physiological, and metabolic characteristics have been identified in metastatic and nonmetastatic cancers. In this setting, high glycolytic activity and poor perfusion (vascular-metabolic mismatch) result in an aggressive tumor phenotype [160]. Finally, advances in the understanding of cancer biology together with developments in diagnostic technologies, and expansion of therapeutic options have all contributed to the concept of personalized cancer care with accurate and specific targeting of cancer cells. Theranostics is the systematic integration of targeted diagnostics and therapeutics. Imaging may select the therapeutic choice and may monitor subsequent changes in the biological characteristics of the tumor [161, 162].

**Fig. 22:**
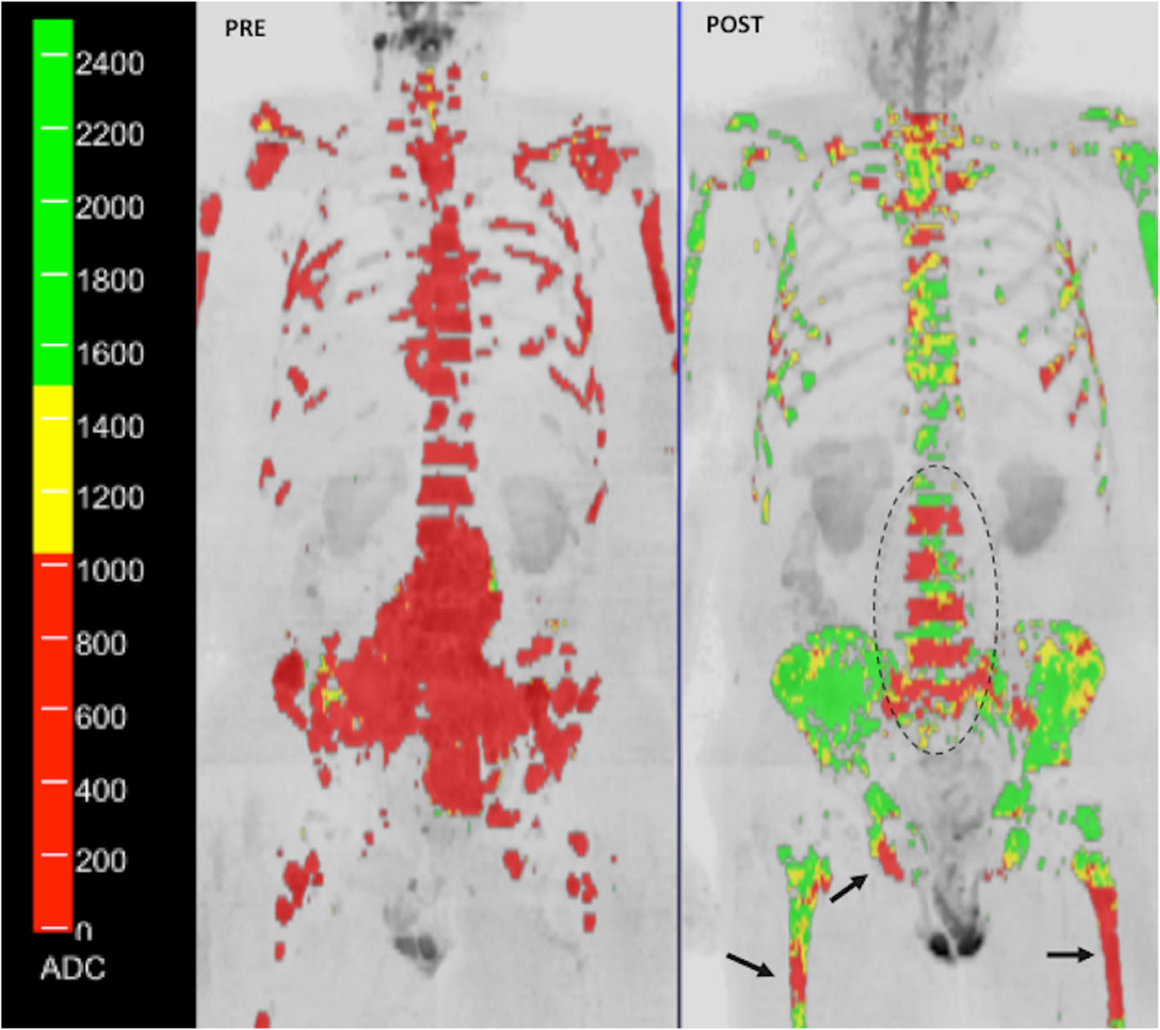
Whole-body DWI evaluation in a 72-year-old man with metastatic prostate cancer treated with (docetaxel + prednisone). A comparison between images pre (left) and posttherapy (right) demonstrated that tumor volume decreased (1280 cm^3^ → 640 cm^3^) and mean ADC moved from 0.7 to 1.61 confirmed an increasing % of voxels at higher ADC values after therapy consistent with reductions in cellularity due to tumor necrosis. However, tumor response was heterogeneous in this patient and there were some anatomical areas that presented a limited tumor response (black dotted circle on lumbar spine and black arrows)

In conclusion, clinical imaging has tremendous potential in the evaluation of a wide spectrum of biological tumor characteristics at all stages of a cancer patient’s management and in drug discovery. Imaging techniques have also the ability to show the spatial and temporal heterogeneity of tumors (Fig. 22). In the time of precision oncology, clinical imaging represents a basic decision-making tool in cancer patients.

## Additional file

Additional file 1: **Table S1.** Imaging Techniques in Tumor Evaluation. (DOCX 36 kb)

## Abbreviations

ADC: Apparent diffusion coefficient
APT: Amide proton transfer
ASL: Arterial spin labeling
BF: Blood flow
BM: Bone marrow
BOLD: Blood oxygenation level dependent
BV: Blood volume
CBF: Cerebral blood flow
CBV: Cerebral blood volume
CEST: Chemical exchange saturation transfer
Cho: Choline
Ci: Citrate
Cr: Creatine
CT: Computed tomography
CuATSM: Diacetyl-bis(N4-methylthiosemi-carbazone) copper(II))
CXCR4: C-X-C motif chemokine receptor 4
D: Tissue diffusivity
D*: Perfusion-related diffusion coefficient
Dapp: Non-Gaussian diffusion coefficient
DCE: Dynamic contrast-enhanced
DKI: Diffusion kurtosis imaging
DSC-MRI: Dynamic susceptibility contrast-enhanced MRI
DTI: Diffusion tensor imaging
DWI: Diffusion-weighted imaging
ECM: Extracellular matrix
EGFR: Epidermal growth factor receptor
EUS: Endoscopic ultrasound
f: Perfusion fraction
FAZA: Fluoroazomycin arabinoside
fD*: Relative perfusion
FDG: Fluorodeoxyglucose
FID: Free induction decay
FLT: Fluorothymidine
FMI: Functional-molecular imaging
FMISO: Fluoromisonidazole
Glut: Glucose transporters
H&N: Head and neck
IAUGC: Initial area under gadolinium curve
IVIM: Intravoxel incoherent motion
Kapp: Apparent kurtosis
kep: Rate constant
*K*^trans^: Transfer constant
Lac: Lactate
Lip: Lipids
LNs: Lymph nodes
MAMMI-PET: Mammography with molecular Imaging PET
MAPK: Mitogen-activated protein kinase
MI: Myo-inositol
MRE: Magnetic resonance elastography
MRI: Magnetic resonance imaging
MRS: Magnetic resonance spectroscopy
MRSI: Magnetic resonance spectroscopic imaging
MT: Magnetization transfer; mechanistic target of rapamycin
MTT: Mean transit time
MVD: Microvessel density
NAA: *N*-acetyl-aspartate
NETs: Neuroendocrine tumors
PCa: Prostate cancer
PET: Positron emission tomography
PI3k: PI3 kinase
PI-RADS v2: Prostate imaging-reporting and data system version 2
ppm: Parts per million
PSMA: Prostate-specific membrane antigen
R2*: Transverse relaxation rate
RCC: Renal cell carcinoma
ROI: Region of interest
SEM: Stretched-exponential model
SI: Signal intensity
SNR: Signal-to-noise ratio
SPECT: Single-photon emission computed tomography
SSTR: Somatostatin receptors
SUV: Standardized uptake value
TIC: Time-intensity curve
TK1: Thymidine kinase-1
TME: Tumor microenvironment
TOLD: Tissue oxygen level dependent
US: Ultrasound
ve: Leakage space fraction
VEGF: Vascular endothelial growth factor
VERDICT: Vascular, extracellular, and restricted diffusion for cytometry in tumors
vp: Fractional plasma volume
WB-DWI MRI: Whole-body diffusion-weighted magnetic resonance imaging
α: Stretching parameter
