# Supplementary material for: How clinical imaging can assess cancer biology
Source: Insights Imaging. 2019 Mar 4;10:28. doi: 10.1186/s13244-019-0703-0 (PMC6399375; doi:10.1186/s13244-019-0703-0)
Supplement: Supplementary file 1 — Table S1. Imaging Techniques in Tumor Evaluation. (DOCX 36 kb) [file 13244_2019_703_MOESM1_ESM.docx]

Additional file 1: **Table S1** Imaging Techniques in Tumor Evaluation

| **Imaging Technique** | **Biological Bases of Imaging Technique** | **Evaluation**  **Parameters obtained** | **Pathophysiological correlation** | **Advantages** | **Disadvantages** |
| --- | --- | --- | --- | --- | --- |
| **Conventional Imaging techniques**  **(CT, US, MRI, mammography)** | Depend on the imaging technique | Morphology: Size, shape, contour  Degree of enhancement with contrast administration  T1, T2, proton density, T2* mapping  Fat quantification | No clear correlation  Non-specific correlation with aggressiveness of some of them | Availability | Unspecific morphologic and structural features without a biological correlation |
| **Dual energy/Spectral CT** | Energy-dependent attenuation profiles of specific materials.  Photoelectric effect increases strongly with increasing effective atomic number, and with decreasing energy of x-rays | Tissue composition  Quantification of attenuation caused by iodine contrast  Calculation of iodine concentrations | Spectral analysis offers opportunity to distinguish clinically relevant materials in tissues (ie, iodine material due to contrast enhancement)  Some correlation with perfusion data in tumors | Evaluation and quantification of contrast material uptake  Possibility of obtaining "virtual" unenhanced images (dose reduction) | Only a few applications are currently well established in clinical practice |
| **Elastography (US or MR-based)** | Tissue hardness | Qualitative evaluation  Quantitative evaluation  Strain ratio | Healthy tissue usually vibrates more than many pathologic processes such as inflammation, fibrosis, and cancer, which induce alterations in tissue stiffness. | Low cost (US)  No ionizing radiation | No definitive histologic correlation  Elastographic measurements are relative |
| **Magnetization transfer MRI** | Differences in magnetization interaction of free water protons and macromolecular-bound protons. | Magnetization transfer ratio | Fibrotic areas due to the presence of collagen | Indirect noninvasive detection of fibrosis | No definitive histologic correlation |
| **DCE-US** | Contrast medium uptake rate in tissues  (Intravascular) | * *Qualitative* evaluation of the type of time/signal intensity curve  * *Semiquantitative* evaluation (maximum upslope, peak enhancement)  * *Quantitative* analysis (depending on mathematical models): Peak-Enhancement, Wash-in-and-Wash-out-AUC, Wash-in Perfusion-Index | Vessel density  Perfusion | Availability  Low toxicity of contrast agents  No ionizing radiation | Permeability cannot be assessed (intravascular contrast agent).  Lack of standardization |
| **Dynamic contrast-enhanced MRI**  **(DCE-MRI)** | Contrast medium uptake rates.  Transfer rates.  Extra-cellular volume  Plasma volume fraction | • *Qualitative:* evaluation of the type of time/signal intensity curve  * *Semiquantitative* *evaluation:* wash-in; washout; time to peak enhancement; etc.  * *Quantitative analysis (based on mathematical models):* Initial area under gadolinium curve (IAUGC); Transfer and rate constants (K^trans^, k_ep_); Leakage space fraction (ve); Fractional plasma volume (vp) | Vessel density Vascular permeability  Perfusion  Extravascular space  Plasma volume | Low toxicity of contrast agents  No ionizing radiation  Versatility in pulse sequences | Complex biological explanation of many parameters  Complex analysis in quantitative models |
| **Dynamic Susceptibility contrast-enhanced MRI**  **(DSC-MRI)** | Contrast medium uptake rate in tissues, which is influenced by:  • Perfusion rates  * Blood volume and blood flow | • *Qualitative* evaluation of the type of time/signal intensity curve  * *Semiquantitative* evaluation:  Relative blood flow, relative blood volume.  Transit time | Vessel density  Perfusion  Vessel size | Low toxicity of contrast agents  No ionizing radiation | Complex biological explanation of many parameters  Complex analysis in quantitative models |
| **Perfusion**  **CT** | Contrast medium uptake rate in tissues, which is influenced by:  • Perfusion & transfer rates  • Extra-cellular volume  • Plasma volume fraction | • *Qualitative* *evaluation*  Time/signal intensity curve  * *Semiquantitative* *evaluation:* Maximum upslope; Peak enhancement; etc.  * *Quantitative analysis (based on mathematical models):* Blood flow (BF); Blood volume (BV); Transit time; Permeability, K^trans^ | Vessel density  Vascular permeability  Perfusion  Plasma volume | Availability | Contrast agent toxicity  Low sensitivity to contrast agents  Exposure to ionizing radiation |
| **ASL (arterial spin labeling)** | Magnetically labeling inflowing arterial blood protons inverting the bulk magnetization of the blood water protons prior to their entry into the tissue of interest. | *Quantitative*: relative changes in cerebral blood volume (CBV), cerebral blood flow (CBF), and mean transit time (MTT) | Perfusion | It does not require intravenous contrast | Technical complexity |
| **BOLD (blood oxygenation level dependent)**  **TOLD (tissue oxygen level dependent)** | Change in relative levels of oxyhemoglobin and deoxyhemoglobin show differential magnetic susceptibility. Deoxygenated hemoglobin is paramagnetic whereas oxygenated hemoglobin is not. | Spin–spin relaxation time (T2) and T2*-weighted signal relaxation  • Transverse relaxation rate (R2*)  • Longitudinal relaxation rate, R1 (=1/T1) | Hypoxia (mainly acute)  Oxygenation  Oxygenation | Identify of moderately to severely hypoxic tumor areas for differential treatment strategies. | Technical complexity  Other variables not related to tissue oxygenation can influence R*2 values  Technical complexity |
| **Imaging techniques**  **based on water diffusion**  **(Diffusion-weighted imaging - DWI)** | **Diffusivity of water** - Monoexponential analysis (**DWI**)  Perfusion component: **Intravoxel incoherent motion (IVIM)**  Structural complexity and heterogeneity: **Diffusion Kurtosis** **Imaging (DKI)**  Heterogeneity: **Stretched-exponential model [SEM]**  **Diffusion tensor imaging (DTI)** | • Apparent diffusion coefficient (ADC)  • Perfusion fraction (f)  • Diffusion (D)  • Perfusion-related diffusion coefficient (D*)  • Relative perfusion (fD*)  • Non-Gaussian diffusion coefficient (Dapp)  • Apparent kurtosis (Kapp): deviations from normal distribution  • Stretching parameter [α]  • Diffusion anisotropy indices  • Fiber orientation mapping | Tissue architecture: cell density & size, extracellular space tortuosity, gland formation, cell membrane integrity, necrosis  Microvessel perfusion  Quantifying the non-gaussianity of any distribution and may evaluate membrane integrity  Anisotropy of tissue structure | Availability  No contrast agents  No ionizing radiation | Technical complexity of advanced techniques (IVIM, DKI, and DTI) |
| **MRS/MRSI** | Atomic nuclei in different electric environments resonate at slightly different frequencies, which are a characteristic of the chemical groups and molecules present | • Ratios between metabolites  • Abnormal Peaks of metabolites  • Absence of normal metabolites | Analysis of metabolic pathways | Specificity  No contrast media | Technical Complexity  Difficult analysis |
| **Positron Emission Tomography (PET)** | Different metabolic pathways depending on the type of radiotracer:  -**Energetic Metabolism** Fluorodeoxyglucose (FDG) | • SUV = standardized uptake value (ratio between tracer uptake and homogeneous distribution of the tracer within the patient). | Glucose uptake | Emission directly proportional to concentration of contrast agent  High sensitivity  Whole body imaging  Relative specificity | High cost  Low spatial resolution (1–2 mm)  No morphological information  Radiation exposure  Short half-life in many radiotracers  -Very short radionuclide agent half-life  No evaluation of permeability  Technical complexity |
|  | -**Tumor Proliferation**  Fluorothymidine (FLT)  Choline (Cho)-PET |  | Activity of thymidine kinase 1  Membranes turnover |  |  |
|  | **-Tumor specific markers** (PSMA, EGFR, SSTR , CXCR4, etc.) |  | The expression of tumor-specific antigens, proteins and surface receptors |  |  |
|  | -**Hypoxia**  (F-MISO), (^64^CuATSM), (F-FAZA) |  | Uptake is influenced by the oxygen level in tissue |  |  |
|  | **-Apoptosis** (Annexin-V) |  | Exposure of phosphatidylserine in the cell membrane |  |  |

ADC: apparent diffusion coefficient; α: stretching parameter; ASL: arterial spin labeling; BF: blood flow; BOLD: blood oxygenation level dependent; BV: blood volume; CBF: cerebral blood flow; CBV: cerebral blood volume; CuATSM: diacetyl-bis(N4-methylthiosemi-carbazone) copper(II)); CXCR4: C-X-C motif chemokine receptor 4; D: tissue diffusivity; D*: perfusion-related diffusion coefficient; Dapp: non-Gaussian diffusion coefficient; DCE: dynamic contrast-enhanced; DKI: diffusion kurtosis imaging; DSC-MRI: dynamic Susceptibility contrast-enhanced MRI; DTI: diffusion tensor imaging; DWI: diffusion-weighted imaging; EGFR: epidermal growth factor receptor; f: perfusion fraction; FAZA: fluoroazomycin arabinoside; fD*: relative perfusion; FDG: fluorodeoxyglucose; FLT: fluorothymidine; FMISO: fluoromisonidazole; IAUGC: initial area under gadolinium curve; IVIM: intravoxel incoherent motion; Kapp: apparent kurtosis; kep: rate constant; K^trans^: transfer constant; MRS: magnetic resonance spectroscopy; MRSI: magnetic resonance spectroscopic imaging; MTT: mean transit time; PET: positron emission tomography; PSMA: prostate-specific membrane antigen; R2*: transverse relaxation rate; SEM: stretched-exponential model; SSTR: somatostatin receptors; SUV = standardized uptake value ; TOLD: tissue oxygen level dependent; ve: leakage space fraction; vp: fractional plasma volume.
